# NADH oxidase of *Mycoplasma hyopneumoniae* functions as a potential mediator of virulence

**DOI:** 10.1186/s12917-022-03230-7

**Published:** 2022-04-02

**Authors:** Fei Hao, Xing Xie, Zhixin Feng, Rong Chen, Yanna Wei, Jin Liu, Qiyan Xiong, Guoqing Shao, Johnson Lin

**Affiliations:** 1 Jiangsu Key Laboratory for Food Quality and Safety-State Key Laboratory Cultivation Base, Ministry of Science and Technology, Key Laboratory for Veterinary Bio-Product Engineering, Ministry of Agriculture and Rural Affairs, Institute of Veterinary Medicine, Jiangsu Academy of Agricultural Sciences, Nanjing, 210014 P.R. China; 2 Discipline of Microbiology, School of Life Sciences, College of Agriculture, Engineering and Science, University of KwaZulu-Natal (Westville campus), Private Bag X 54001, Durban, 4000 South Africa; 3 Hubei Key Laboratory of Animal Nutrition and Feed Science, Wuhan Polytechnic University, Wuhan, 430023 P.R. China

**Keywords:** *Mycoplasma hyopneumoniae*, NADH oxidase, Adhesion, Virulence factor

## Abstract

**Background:**

*Mycoplasma hyopneumoniae* (*M. hyopneumoniae*) is the etiological agent of enzootic pneumonia, a highly infectious swine respiratory disease that distributed worldwide. The pathogenesis and virulence factors of *M. hyopneumoniae* are not fully clarified. As an important virulence factor of bacteria, nicotinamide adenine dinucleotide (NADH) oxidase (NOX) participates in host-pathogen interaction, however, the function of NOX involved in the pathogenesis of *M. hyopneumoniae* is not clear.

**Results:**

In this study, significant differences in NOX transcription expression levels among different strains of *M. hyopneumoniae* differed in virulence were identified*,* suggesting that NOX may be correlated with *M. hyopneumoniae* virulence. The *nox* gene of *M. hyopneumoniae* was cloned and expressed in *Escherichia coli*, and polyclonal antibodies against recombinant NOX (rNOX) were prepared. We confirmed the enzymatic activity of rNOX based on its capacity to oxidize NADH to NAD^+^. Flow cytometry analysis demonstrated the surface localization of NOX, and subcellular localization analysis further demonstrated that NOX exists in both the cytoplasm and cell membrane. rNOX was depicted to mediate adhesion to immortalized porcine bronchial epithelial cells (hTERT-PBECs). Pre-neutralizing *M. hyopneumoniae* with anti-rNOX antibody resulted in a more than 55% reduction in the adhesion rate of high- and low-virulence *M. hyopneumoniae* strains to hTERT-PBECs. Moreover, a significant difference appeared in the decline in CCU_50_ titer between virulent (168) and virulence-attenuated (168L) strains. NOX not only recognized and interacted with host fibronectin but also induced cellular oxidative stress and apoptosis in hTERT-PBECs. The release of lactate dehydrogenase by NOX in hTERT-PBECs was positively correlated with the virulence of *M. hyopneumoniae* strains.

**Conclusions:**

NOX is considered to be a potential virulence factor of *M. hyopneumoniae* and may play a significant role in mediating its pathogenesis.

**Supplementary Information:**

The online version contains supplementary material available at 10.1186/s12917-022-03230-7.

## Background

Porcine respiratory diseases, which are characterized by high morbidity and mortality, medications cost, feed efficiency and growth rate decreasing , and lower quality of pig carcasses, led to a considerable impact on the pig industry [1]. Enzootic pneumonia (EP) is a highly infectious and prevalent, global, chronic, swine respiratory disease that is caused by *Mycoplasma hyopneumoniae* (*M. hyopneumoniae*) infection, which is characterized by nonproductive, dry coughing, severe respiratory distress, growth retardation, and inefficient feed conversion [2]. *M. hyopneumoniae* is considered to be one of the primary pathogens involved in porcine respiratory disease complex (PRDC) [1, 3]. Infected pigs often develop secondary infections involving a range of other pathogens, such as porcine reproductive and respiratory syndrome virus (PRRSV), porcine circovirus (PCV), and *Pasteurella multocida* [4]. This comorbidity phenomenon hinders proper diagnosis of specific diseases due to complicated symptomatic manifestations, which leads to an increased mortality rate in infected pigs. Thus, the mechanisms underlying *M. hyopneumoniae* pathogenesis are not fully understood. Some reports have suggested that the onset of EP depends on virulence factors of *M. hyopneumoniae* that enable the pathogen to override host defense mechanisms by inducing a biochemical host response that promotes cell adhesion and immunomodulation [5]. Therefore, the identification and functional analysis of novel virulence-related genes would be helpful in building a more complete understanding of *M. hyopneumoniae* pathogenesis.

Several studies have also demonstrated that mycoplasma metabolic enzymes play more than a basic role by possibly serving as an important determinant of pathogenicity [6–9]. Among them, nicotinamide adenine dinucleotide (NADH) oxidases (NOX) of *Mycoplasma bovis* have shown possible participation in virulence [7]. Moreover, comparative proteomics findings have demonstrated significant variations in the expression of NOX among various *M. hyopneumoniae* strains that differed in degree of virulence [10, 11]. NOX belongs to the largest group of enzyme oxidoreductases, which function in catalyzing the reduction of reactive superoxide species into H_2_O_2_ or H_2_O by simultaneously oxidizing NADH to NAD^+^. Various organisms contain these enzymes, which play important roles in regulating cellular redox and osmotic pressure balance to maintain normal cell growth and development. In addition to its effect on metabolic processes, NOX also plays a vital role in bacterial oxidative stress response, bacterial membrane formation, and virulence regulation [12]. More importantly, NOX of *M. hyopneumoniae* has already been reported to reside on the cell surface and is suspected to bind to fibronectin, heparin, actin, and plasminogen [9, 13]. Proteins located in multiple subcellular locations with different functions are called “moonlighting” proteins [14]. Several dozen of these proteins have been identified as ubiquitous intracellular enzymes or intracellular/ surface moonlighting proteins (ISMPs), which function in essential cellular processes [14]. Thus, NOX of *M. hyopneumoniae* may be linked to its virulence.

As a novel virulence factor candidate identified in this study, NOX was found in both the cytoplasm and cell membrane of *M. hyopneumoniae*. NOX specifically adhered to immortalized porcine bronchial epithelial cells (hTERT-PBECs) and recognized host fibronectin. NOX not only shows its enzyme characteristics but also induces obvious cytotoxicity, oxidative stress damage, and apoptosis in hTERT-PBECs. Our findings support the notion that NOX may be a potential novel virulence factor of *M. hyopneumoniae*, and these findings will provide new ideas and theoretical support for studying the pathogenic mechanisms of *M. hyopneumoniae* and other mycoplasmas.

## Results

### Significant differences in the transcription level of *nox* genes between high and low virulence strains of *M. hyopneumoniae*

**Fig. 1:**
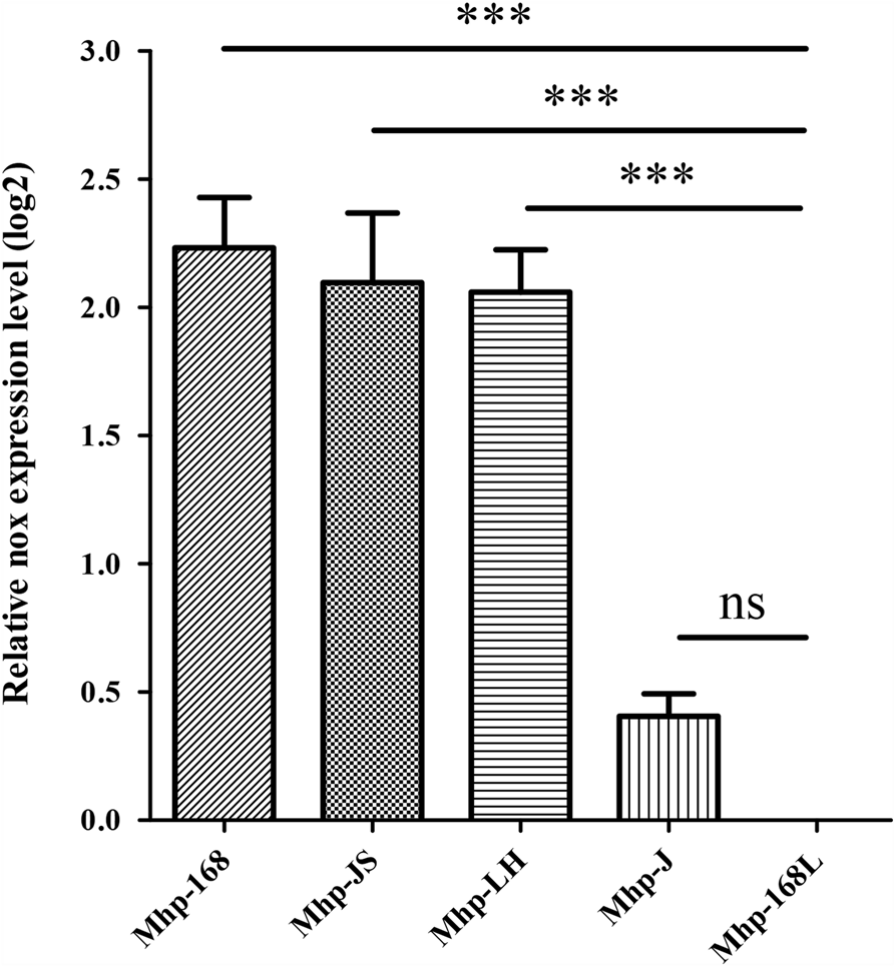
Relative expression levels of *nox* genes involved in different *M. hyopneumoniae* (Mhp) compared to Mhp strain 168L (set to 1). Gene expression was determined by quantitative real-time PCR analysis. Error bars represent standard deviations from three independent experiments (***: *p<*0.001; ns: *p* >0.05)

To investigate mRNA expression differences of the *nox* gene between *M. hyopneumoniae* (Mhp) strains of differing virulence, relative quantitative reverse transcription polymerase chain reaction (RT-PCR) was performed. As shown in Fig. 1, the mRNA expression levels of the *nox* gene were significantly upregulated in the highly virulent strains Mhp 168, LH and JS compared to the virulence-attenuated strain Mhp 168L (*p* < 0.001). In addition, the mRNA expression levels of the *nox* gene in low virulent strain J were higher than that in the virulence-attenuated strain Mhp 168L, but with no significant difference (*p* > 0.05).

### Bioinformatic analysis, protein expression, and enzymatic activity of *M. hyopneumoniae* NOX

Eighteen amino acid sequences of NOX from *M. hyopneumoniae* were retrieved from the NCBI and UniProt Protein databases (Genbank accession numbers were shown in supplementary file), and the homologies among them were analyzed. All sequences were aligned using the CLUSTAL W program, as shown in supplementary file Fig. S1A. Molecular Evolutionary Genetics Analysis version 10 (MAGE10) was used for phylogenetic inference according to the neighbor-joining criterion. The robustness of the hypothesis was evaluated with 1000 nonparametric bootstrap analyses (Fig. S1B). The *nox* gene of *M. hyopneumoniae* contains an open reading frame of 1383 base pairs (bp) encoding 460 amino acids. The results of comparative sequence analysis indicated that the amino acid sequences of NOX among all *M. hyopneumoniae* strains shared more than 99.34% homology with each other. It also showed that NOX is a highly conserved protein, and there were few differences among various *M. hyopneumoniae* strains. Based on codon optimization, the prokaryotic expression plasmid pET-21a-nox was constructed and expressed in *Escherichia coli* BL21 (DE3). Expression of purified recombinant NOX protein (rNOX) with a size of 50.6 KDa was shown in panel “a” of Fig. S2. As shown in panel “b” of Fig. S2, the Western blot analysis results showed that the prepared polyclonal antibody could specifically recognize the recombinant protein rNOX. Then, the enzymatic activity of rNOX was determined after purification and renaturation. The enzymatic specific activity of the rNOX protein was determined to be 25.23 IU/mg.

### NOX located both on the surface and in the cytoplasm of *M. hyopneumoniae* cells

**Fig. 2:**
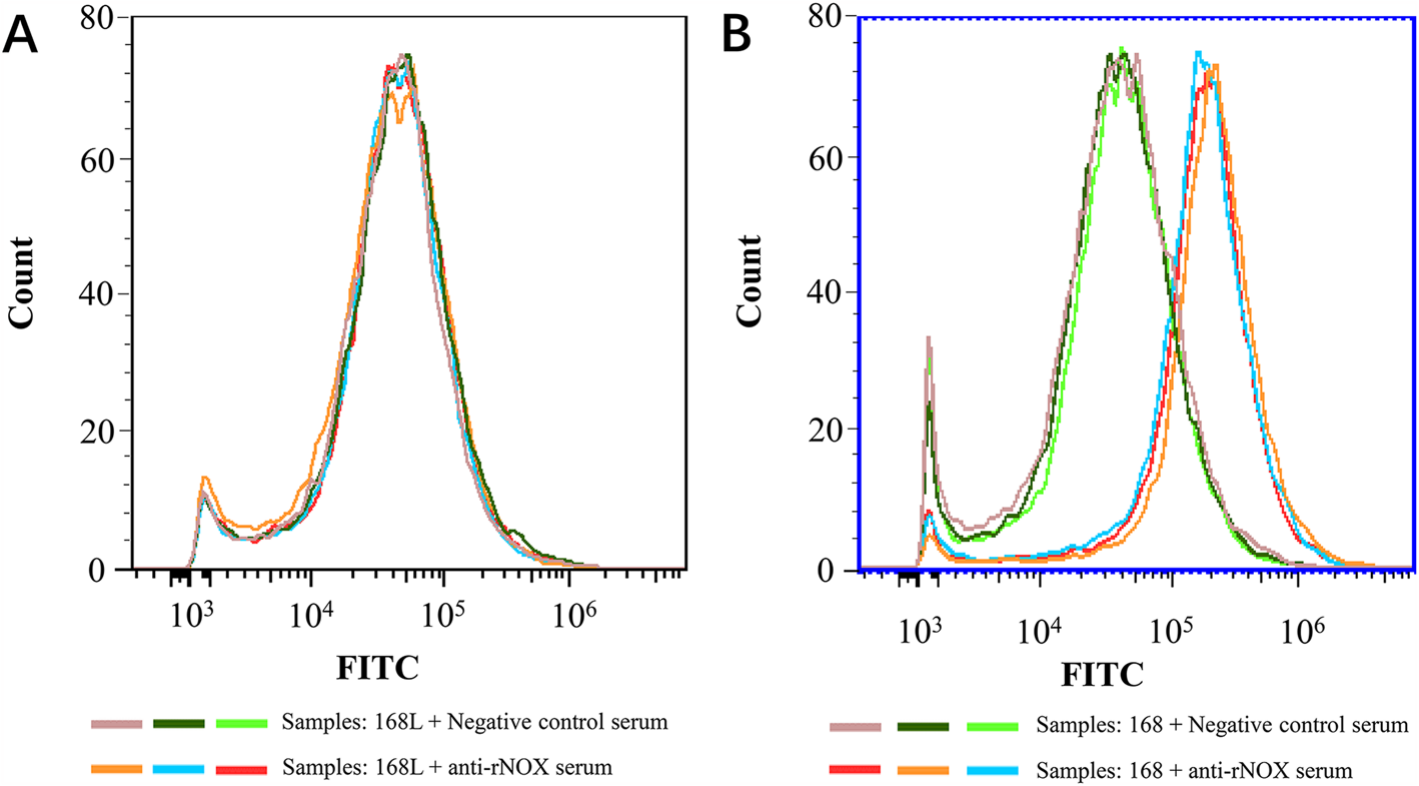
Detection of surface-exposed NOX by flow cytometry. Comparison of fluorescence intensity of Mhp strains differed in virulence when treated with anti-NOX serum and negative serum. A: Negative control, Mhp strain 168L treated with pre-immune serum; Mhp strains 168L treated with anti-Mhp NOX serum. B: Negative control, Mhp strain 168 treated with pre-immune serum; Mhp strains 168 treated with anti-Mhp NOX

To investigate whether NADH oxidase is present on the surface of *M. hyopneumoniae*, flow cytometry analysis was performed. The results showed that the outer membrane-localized NOX was surface-accessible to NOX-specific antibodies in Mhp strains 168 and 168L, initially suggesting that NOX antigen was present on the bacterial cell surface of *M. hyopneumoniae* strains. There was no significant difference in the mean fluorescence intensity (MFI) between the *M. hyopneumoniae* strain 168L treated with anti-rNOX serum and strain 168L incubated with pre-immune serum (Fig. 2A), whereas the MFI of strain 168 treated with anti-rNOX serum was approximately 10-fold higher than that of strain 168 treated with pre-immune serum (Fig. 2B), suggesting that NOX may correlate with the virulence of *M. hyopneumoniae* strains.

**Fig. 3:**
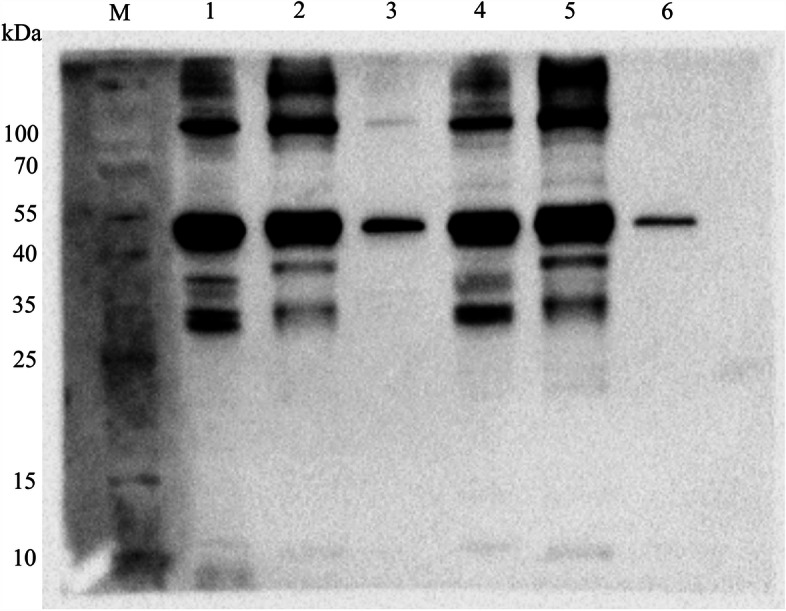
Subcellular localization of NADH oxidase in Mhp strains with different degrees of Virulence. Lane M is pre-stained protein mass markers, Lane 1, whole cell lysates of Mhp strain 168; Lane 2, cytoplasmic proteins of Mhp strain 168; Lane 3, membrane proteins of Mhp strain 168; Lane 4, whole cell lysates of Mhp strain 168L; Lane 5, cytoplasmic proteins of Mhp strain 168L; Lane 6, membrane proteins of Mhp strain 168L

The whole-cell, membrane, and cytoplasmic fractions of *M. hyopneumoniae* were prepared for Western blot to determine the subcellular localization of NOX using rabbit anti-rNOX serum. The results showed that NOX protein was distributed in the *M. hyopneumoniae* cytoplasm and cell membrane, NOX was more distributed in the cytoplasm (Fig. 3). Moreover, the results also showed that the content of NOX in the membrane of virulent strain 168 was higher than that in virulence-attenuated strain 168L.

### rNOX binds hTERT-PBECs and anti-rNOX serum inhibits *M. hyopneumoniae* adherence to hTERT-PBECs.

**Fig. 4:**
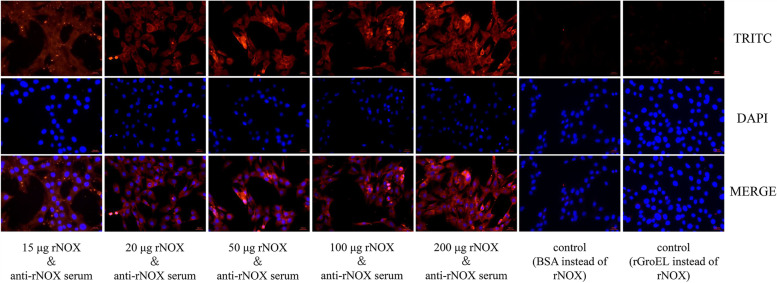
rNOX adhesion assay with IFA. TRITC, antibody reactivity to IgG observed with TRITC-conjugated anti-rabbit IgG antibody (orange red); DAPI, nuclei of all cells were stained by DAPI reagent (blue); an overlay of the images is shown in the column labeled ‘‘Merge’’. Captured images were observed at 400× magnification. The red bar represents the scale: 100 μm

Indirect immunofluorescence assays (IFAs) were used to determine whether *M. hyopneumoniae* NOX could specifically adhere to the surface of hTERT-PBECs. The IFA results revealed significant punctate fluorescence on the cell surface of hTERT-PBECs incubated with rNOX; however, no specific punctate fluorescence was found around 6-diamidino-2-phenylindole (DAPI)-stained cell nuclei in negative controls (Fig. 4). Moreover, the results show that there was dose-dependent binding of rNOX to hTERT-PBECs, and the upper limit of concentration to saturate the host cell surface was 200 μg. However, no adhesion was observed when the lower limit concentration of rNOX was below 15 μg. Thus, it appears that rNOX may bind specifically to hTERT-PBEC membranes.

**Fig. 5:**
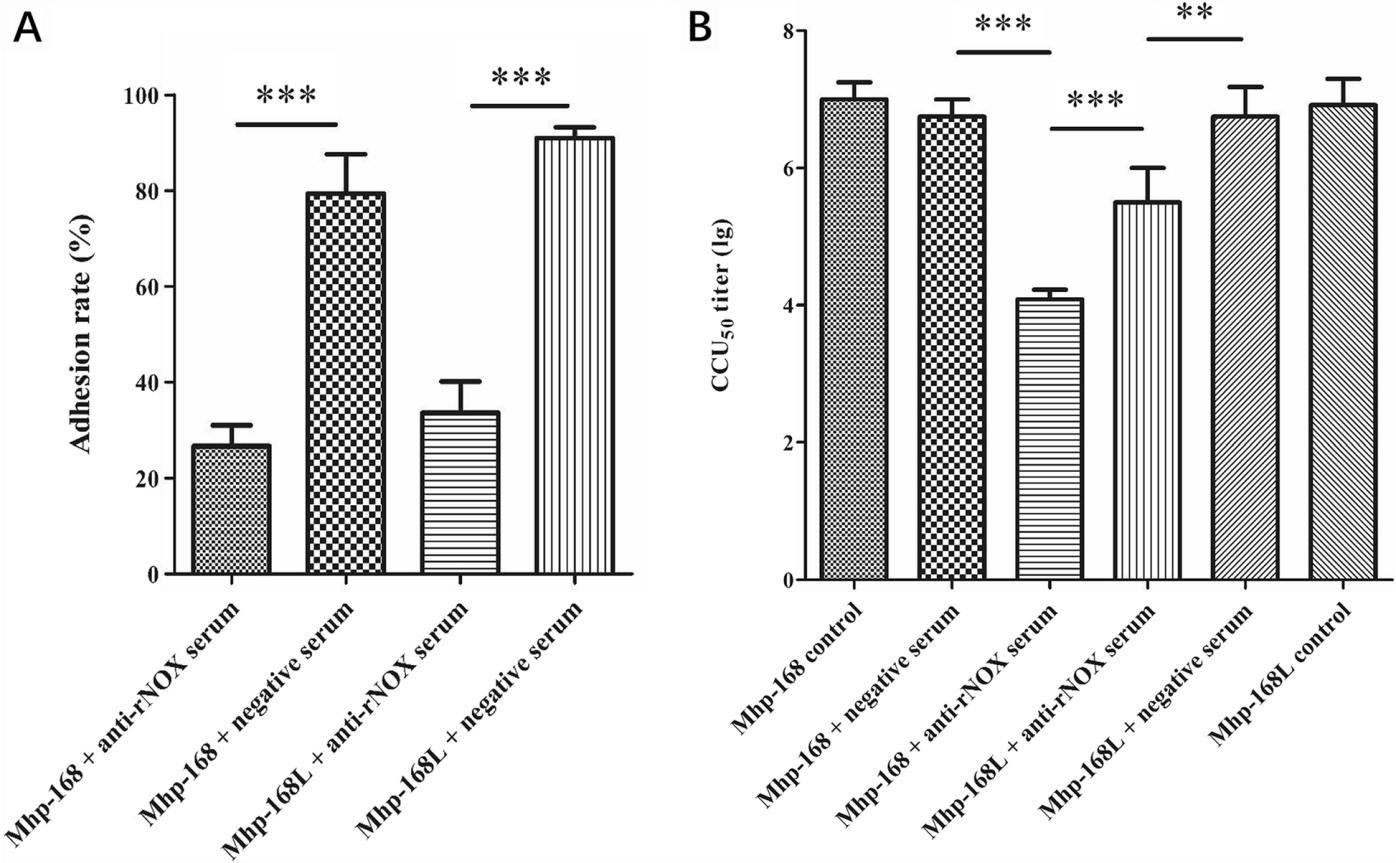
rNOX adhesive inhibition assays with real-time PCR and CCU_50_ assay. **A** Adhesion rates were calculated by real-time PCR for Mhp bacterial counting. Adhesion rate = (number of Mhp antigens collected from infected cells incubated with the anti-rNOX serum/ number of Mhp antigens collected from infected cells in the group incubated with the pre-immune sera) × 100. Quantitative real-time PCR analysis was performed and expressed as log10 DNA copy number per mL of Mhp strain-infected cell distribution against P97. Data are presented as the mean ± SD of three independent experiments (***: *p<*0.001). **B** Mhp titers were quantified using a CCU_50_ assay. Data are presented as the mean ± SD of three independent experiments (***: *p<*0.001; **: *p<*0.01).

To further evaluate the role of NOX in *M. hyopneumoniae* adhesion to hTERT-PBECs, an antibody inhibition assay was performed. Compared with the control group treated with negative serum, the anti-rNOX serum could reduce the adherence of *M. hyopneumoniae* (both strains 168 and 168L) to hTERT-PBECs. The adherence degree was shown as the adhesion rate compared with the adherence of *M. hyopneumoniae* in the absence of antibody. Incubation with anti-rNOX antibody resulted in 52.73% (strain 168) and 57.47% (strain 168L) (*p* < 0.001) reductions in the adhesion rate of *M. hyopneumoniae* to hTERT-PBECs (Fig. 5A). In addition, pre-neutralizing *M. hyopneumoniae* with anti-rNOX antibody resulted in a 2.67 (strain 168; *p* < 0.001) and 1.25 (strain 168L; *p* < 0.01) reduction in the 50% color changing unit (CCU_50_) of *M. hyopneumoniae*, and there was a significant difference in CCU_50_ titer decline between virulent strain 168 and virulence-attenuated strain 168L (*p* < 0.001) (Fig. 5B). This result further confirmed that *M. hyopneumoniae* NOX may function as a potential adhesion factor, playing an essential role in the adherence of *M. hyopneumoniae* to host cells.

### Identification of rNOX binding ligands

**Fig. 6:**
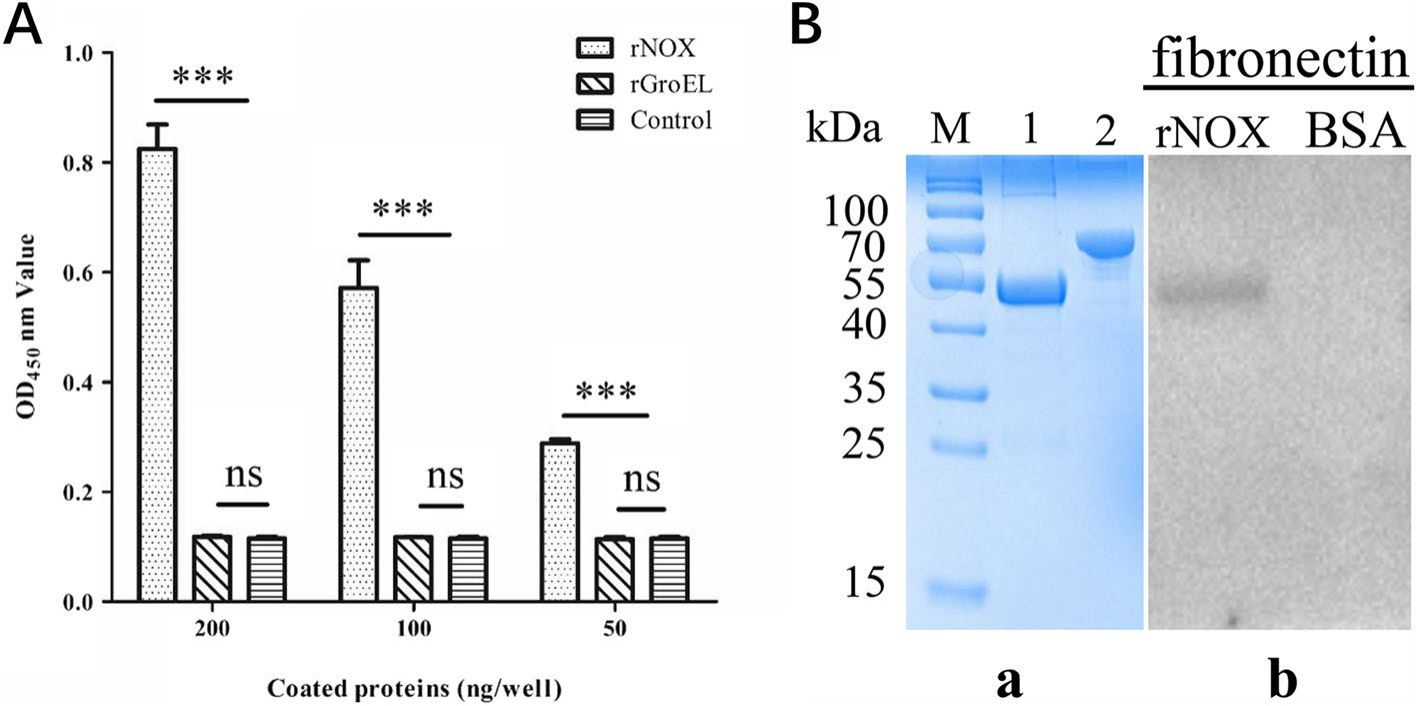
**A** rNOX, rGroEL, and PBS were applied to examine fibronectin activity with indirect ELISA. *** represents an extremely significant difference (*p* < 0.001), while “ns” represents no significant difference between groups (*p* >0.05). **B** “a,” SDS–PAGE analysis of purified recombinant protein rNOX and BSA. Lane M is pre-stained protein mass markers; Lane 1: purified recombinant protein rNOX; Lane 2: BSA. The gel in this figure was cropped and the full-length gel was presented in Supplementary FigureS3. “b,” Mhp NOX with fibronectin interaction analysis by far Western blot. Lane rNOX: PVDF membrane with transferred Mhp NOX protein incubated with fibronectin and the anti-fibronectin antibody. Lane BSA: PVDF membrane with transferred BSA (negative control) incubated with fibronectin, and the anti-fibronectin antibody. The blots in this figure were cropped and the full-length blots were presented in Supplementary FigureS4.

To determine whether hTERT-PBEC components interact with NOX we conducted ELISA and Far-Western blot analyses to examine the interaction between *M. hyopneumoniae* NOX and fibronectin. ELISA results showed that rNOX had a stronger binding ability to fibronectin than to phosphate buffered saline (PBS) and the unrelated protein (heat shock protein Hsp60 of *Aeromonas hydrophila*, rGroEL) (Fig. 6A). The ELISA results also indicated that rGroEL has no binding ability to fibronectin compared to that of the PBS control. As shown in panel “b” of Fig. 6B, a corresponding band was observed in the reactions of rNOX to the anti-rNOX antibody (positive control) before finally reacting to fibronectin, while no specific reaction was observed in the negative control (bovine serum albumin, BSA). All these results showed that *M. hyopneumoniae* NOX could specifically bind to and exhibit high affinity for fibronectin.

### rNOX induced cytotoxicity, oxidative-stress damage, and apoptosis of hTERT-PBECs

**Fig. 7:**
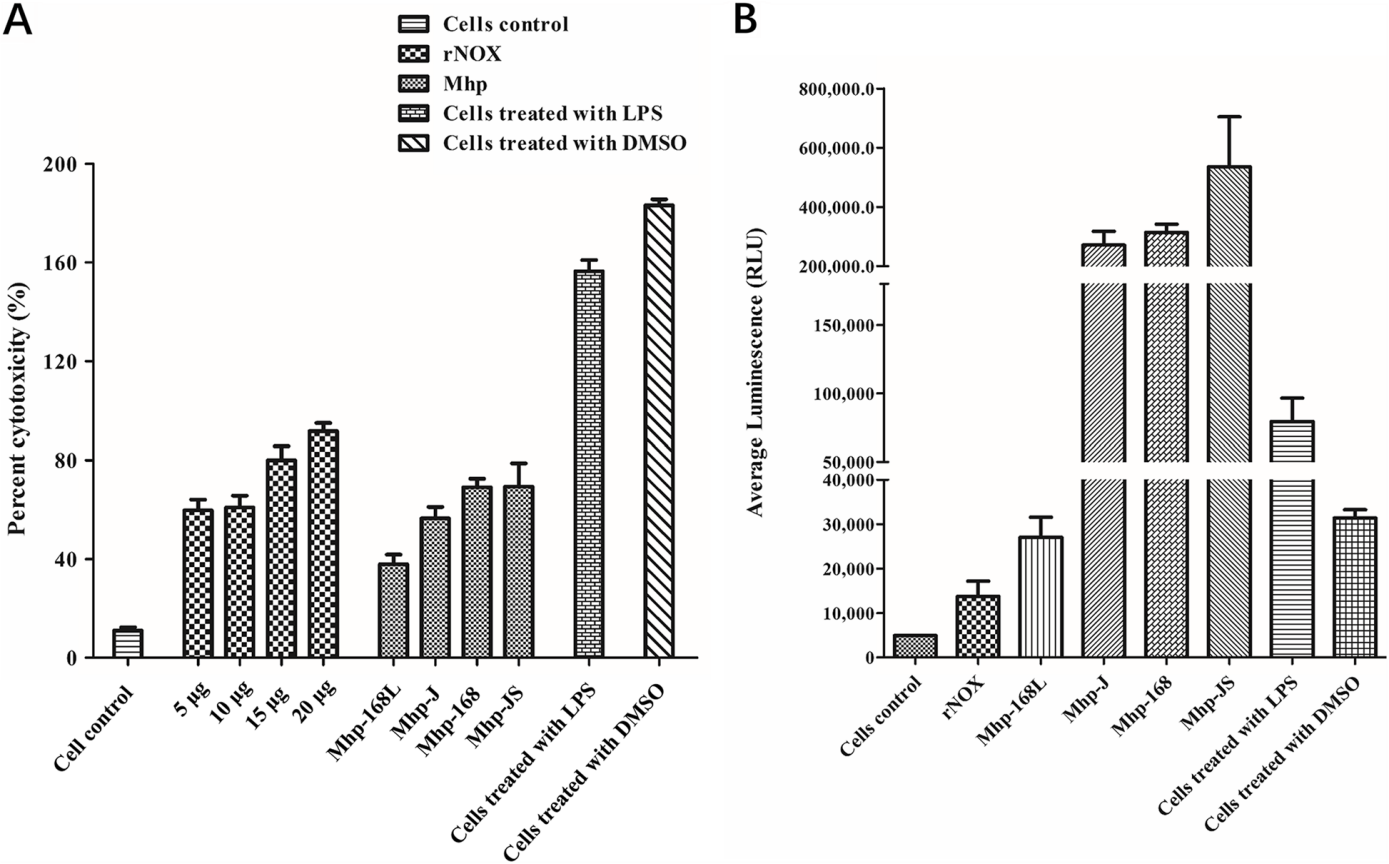
Cytotoxicity and oxidative stress of hTERT-PBECs induced by rNOX. **A** CytoTox 96® Non-Radioactive Cytotoxicity Assay of hTERT-PBEC cytotoxicity after treatment with different concentrations of rNOX and various strains of Mhp with varying degrees of virulence (strains JS, 168, J and 168L). **B** ROS-Glo™ H2O2 assay of host cell cellular oxidative stress after treatment with rNOX and Mhp strains JS, 168, J and 168L

As shown in Fig. 7A, rNOX induced 59.73%, 60.82%, 79.99%, 91.71% cytotoxicity at concentrations of 5 μg, 10 μg, 15 μg and 20 μg, respectively. At the same time, the cytotoxicity of strain infection controls of *M. hyopneumoniae* infection was 37.86% (168L), 56.45% (J), 69.10% (168) and 69.35% (JS). The cells treated with lipopolysaccharides (LPS) and dimethyl sulfoxide (DMSO) (positive controls) induced cytotoxicity at 156.43% and 183.14%, respectively.

rNOX could induce oxidative stress damage in hTERT-PBECs, as measured by the average luminescence expressed in relative luminescence units (RLUs). As shown in Fig. 7B, 20 μg rNOX induced hTERT-PBECs to produce 13735 RLU of reactive oxygen species (ROS), which was higher than that of the negative control. At the same time, there were 79386 RLU and 31405 RLU production of ROS in the positive control (cells treated with LPS and DMSO), respectively. Moreover, rNOX could not induce oxidative stress damage in hTERT-PBECs treated with low concentrations of rNOX (< 20 μg, data not shown). Up to 20 μg rNOX or more could induce oxidative stress damage in hTERT-PBECs. The ROS release from hTERT-PBECs induced by the *M. hyopneumoniae* strain was 27045 RLU (168L), 271068 RLU (J), 313941 RLU (168), and 535685 RLU (JS), indicating that virulence-attenuated *M. hyopneumoniae* strain 168L-induced ROS release against cells was significantly lower than the three virulent strains (JS, 168 and J).

**Fig. 8:**
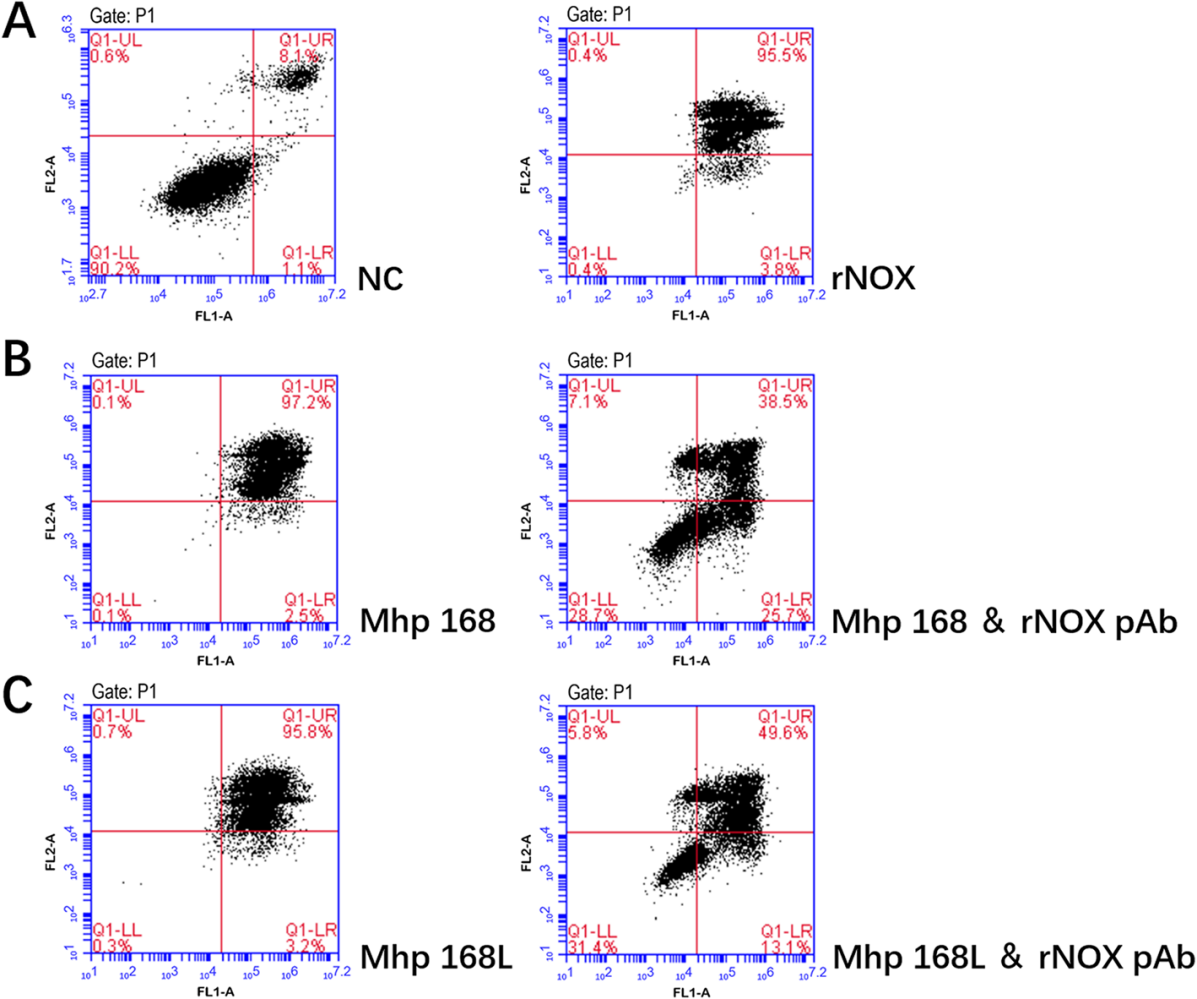
The apoptosis of hTERT-PBECs induced by rNOX and different virulent Mhp strains was assessed by flow cytometry using the annexin-V-FITC/PI double staining method 12 h post-infection. **A**: Negative control hTERT-PBECs cultured for 12 h in DMEM/F12 medium without FBS and growth factors before double staining; rNOX, the apoptosis rate of recombinant rNOX protein-infected hTERT-PBECs 12 h post-infection before double staining. **B**: Highly virulent Mhp strain 168 infected with hTERT-PBECs for 12 h treated without and with rNOX polyclonal antibody. **C**: Low virulent Mhp strain 168L infected with hTERT-PBECs for 12 h treated without and with rNOX polyclonal antibody

Moreover, flow cytometry results indicated that rNOX could also induce apoptosis in hTERT-PBECs, and the late-stage apoptosis rate was upregulated by 87.4% compared with the negative control group. The late-stage apoptosis induced by *M. hyopneumoniae* strains 168 or 168L in hTERT-PTECs was significantly decreased when rNOX was blocked with an anti-rNOX antibody. The late-stage apoptotic cell percentage (upper right quadrant) decreased by 58.7% and 46.2%, respectively (Fig. 8).

## Discussion

Mycoplasmas are the simplest self-replicating organisms and are thought to have evolved from gram-positive bacteria through reductive evolution [15]. As a result of their limited biosynthetic and metabolic capabilities, mycoplasmas rely on infected host cells for nutrition and possibly other metabolic needs [16]. Bacterial pathogens, including *M. hyopneumoniae* [17], form a biofilm by adhering to the proximal, solid-phase vector, which enhances their transmission. Adhesion ability is a crucial factor that appears to correlate with bacterial virulence. Fimbriae and cell wall components are the usual constituents involved in adhesion. However, because the dramatic genomic downsizing of mycoplasmas has resulted in the loss of the cell wall, surface lipoproteins play a vital role in their adhesion to, and invasion of hosts as well as their ability to evade host immune responses [13, 17, 18]. Apart from surface lipoproteins, metabolic enzymes are also known to play a role in host-pathogen interactions [8]. Furthermore, beyond the role of oxidoreductase in metabolism, it appears that NOX may also play a role in mediating the virulence of bacterial pathogens [7, 12].

*M. hyopneumoniae* is known as a host-specific pathogen that infects only pigs including domestic pigs and wild boars [2]. Many factors in the virulence of *M. hyopneumoniae* have been shown to play roles in adhesion, cellular invasion, and intracellular proliferation. However, the molecular mechanisms of infection and pathogenesis of *M. hyopneumoniae* have remained unclear [2, 5, 19]. Previous comparative proteomics studies have demonstrated that NOX was overexpressed in the high virulent 7448 strain compared to nonpathogenic *M. flocculare* [15]. Here, in this study, mRNA expression levels of the *nox* gene in the pathogenic 168, LH, and JS strains were significantly upregulated compared to those in the virulence-attenuated strain 168L. This result preliminarily suggests that NOX may have a correlation with the virulence of *M. hyopneumoniae*. Further analyses using flow cytometry showed that NOX is present on the surface of *M. hyopneumoniae*, which is consistent with a previous report [9]. Subcellular localization analysis further indicated that NOX is distributed in both the *M. hyopneumoniae* cytoplasm and the cell membrane and, compared with the low virulence strain 168L, fluorescence intensity of the pathogenic strain 168 was greater by a factor of 10. The results of subcellular localization analysis also showed that the content of NOX in the membrane of virulent strain 168 is higher than that in virulence-attenuated strain 168L. All these results further indicated that NOX may be correlated with virulence of *M. hyopneumoniae* strains.

As is the case with most ISMPs, further study is needed to understand NOX production, attachment to cell surfaces, and performance in extracellular functions. Various hypotheses have been suggested regarding these processes [14]. For example, some ISMPs are produced and found to be associated with cell surfaces. An increase in extracellular pH has been shown to cause some *Lactobacillus crispatus* ISMPs to be released from cell surfaces [20]. In most cases, the components of cell surfaces (e.g.: proteins, or lipids, etc.) that bind ISMPs remain unknown. However, recent studies have shown that extracellular enolase is bound to a rhamnose residue in the cell membrane of mycoplasmas [21], while enolase and glyceraldehyde-3-phosphate dehydrogenase (GAPDH) can covalently bind to lipoteichoic acid on *L. crispatus* [20], and NADH-dependent flavin oxidoreductase (NFOR) of *M. hyopneumoniae* has been shown to interact with host fibronectin and plasminogen [22]. Although it is still unclear how NOX acts as an enzyme in the cell matrix, stimulating adhesion on cell surfaces, the investigations described here provide findings that can help to guide further study.

The adhesion of microorganisms, including mycoplasmas, to their host cells is a crucial step in their colonization and subsequent infection of a host. *M. hyopneumoniae* is commonly found on the mucosal surfaces of swine trachea, bronchi and bronchioles, where it can induce ciliostasis and loss of cilia [23]. The first stage of pathogenesis is adhesion to cilia in epithelial cells of the respiratory mucosa by means of adhesins. In this study, we found that NOX can adhere to hTERT-PBECs which were established in our previous work [24]. In addition, *M. hyopneumoniae* pre-neutralized with polyclonal antiserum to rNOX significantly reduced the adherence of *M. hyopneumoniae* to host cells. Fibronectin has been widely studied and determined to be a prevalent extracellular matrix protein that can form a molecular bridge between a pathogen and host cellular receptors [25]. Several fibronectin-binding bacterial proteins were found to mediate adhesion and subsequent invasion of bacteria into host cells through binding to fibronectin [26]. Therefore, fibronectin-binding proteins play a key role in the pathogenic process of bacteria. NOX of *M. hyopneumoniae* has already been shown to reside on the cell surface and is reported to bind to fibronectin, heparin, actin and plasminogen [9, 13]. Similarly, in this study, we confirmed that rNOX has a high affinity for, and can bind specifically to fibronectin. It may be possible that fibronectin functions as a primary receptor of NOX, thus mediating the adhesion process of *M. hyopneumoniae*.

Lactate dehydrogenase (LDH) is a stable cytoplasmic enzyme that converts lactate to pyruvate. Measurement of LDH release by damaged cells offers a method for detecting cell viability from the perspective of cell membrane integrity [27]. We found that NOX can induce dose-dependent host cell cytotoxicity, and LDH release of NOX in host cells is positively correlated with the virulence of various *M. hyopneumoniae* strains. ROS are generated during many cellular processes, for example aerobic respiration, but most ROS are neutralized by antioxidants. Bacterial or viral infections, inflammatory reactions, ionizing radiation, and many chemical drugs also produce a considerable amount of ROS [28, 29]. In these processes, if the level of ROS exceeds the neutralizing limit of antioxidant proteins, so-called “oxidative stress” will result. Apoptosis refers to the autonomous and orderly death of cells controlled by genes to maintain the stability of the overall intracellular and extracellular environment. Unlike cell necrosis, apoptosis is not a passive process but a phenomenon that induces autologous injury through a series of signal activations, protein expressions, and other regulations [30]. Research has demonstrated that ROS deposition is considered to be a direct cause of apoptosis because ROS have strong cytotoxicity to cells [31]. When intracellular ROS rise sharply, they stimulate oxidative stress and apoptosis [32]. We found that rNOX could induce obvious oxidative stress damage in hTERT-PBECs. In addition, virulence-attenuated *M. hyopneumoniae* strain 168L induced ROS release that was significantly lower than the other three virulent strains (JS, 168 and J). More importantly, rNOX alone produced significantly lower levels of oxidative stress than the attenuated 168L strain. This result may indicate that *M. hyopneumoniae* contains other factors that lead to higher oxidative stress in infected host cells. Moreover, flow cytometry results indicated that rNOX could induce apoptosis in hTERT-PBECs as well, and the late-stage apoptosis rate was upregulated by 87.4% compared with the negative control group (4.9%) after 12 h. Late-stage apoptosis induced by high virulence *M. hyopneumoniae* strain 168 or low virulence strain 168L in porcine bronchial epithelial cells was significantly decreased when rNOX was blocked with anti-rNOX antibody, with the late-stage apoptotic cell percentage decreasing by 58.7% and 46.2%, respectively. In conclusion, the above results further indicate that NOX, like the positive control (various *M. hyopneumoniae* strains), may function as a mediator of virulence in *M. hyopneumoniae* strains.

## Conclusions

The study showed that significant differences exist in the transcription levels of *nox* genes between high and low virulence strains, suggesting that NADH oxidase may be related with *M. hyopneumoniae* virulence. In addition, NADH oxidase was found in both the cytoplasm and cell membrane of *M. hyopneumoniae* and it has strong binding affinity with fibronectin. Besides, NADH oxidase had the ability to adhere to immortalized porcine bronchial epithelial cells and it could also induce host cell cellular oxidative stress, cytotoxicity, and apoptosis. In conclusion, NADH oxidase is considered to be a potential virulence factor candidate of *M. hyopneumoniae*, which may play a pivotal role in mediating its pathogenesis.

## Methods

### Bacterial strains, cell lines, and growth/ culture conditions

All *M. hyopneumoniae* strains in this study were thawed from frozen stocks and subcultured three times before use. *M. hyopneumoniae* strain J (ATCC25934), which was subcultured once to establish frozen stocks from ATCC. Strain 168 was isolated from a pig exhibiting typical characteristics of EP in Gansu Province, China [33]. This field strain was gradually attenuated by continuous subculturing to the 350^th^ passage through KM2 cell-free liquid medium (modified Friis medium) containing 20% (v/v) swine serum at 37 °C, to yield strain 168L [34]. Thus, the strain 168L used in our work was derived from subculture 353. Strain JS is a virulent strain that can induce typical characteristics of EP with a lung lesion score of approximately 15, as described previously [35]. Strain LH was also a virulent clinical strain isolated in our lab and capable of inducing typical characteristics of EP (GenBank accession number: CP079799) [22]. These five *M. hyopneumoniae* strains were cultured in modified Friis’ medium designated KM2 cell-free medium containing 20% (v/v) swine serum (produced in our lab from a clean snatch-farrowed, porcine-colostrum-deprived (sF-pCD) piglet after irradiation sterilization) at 37 °C in a humidified incubator. Titers of *M. hyopneumoniae* strains were quantified using a CCU_50_ assay [36], which was modified based on the color changing unit (CCU) assay [37] in culture medium and confirmed by quantitative PCR.

Immortalized porcine bronchial epithelial cells (hTERT-PBECs) were established for *M. hyopneumoniae* infection *in vitro* by our laboratory [24]. The cells were grown in Dulbecco's modified Eagle’s medium: nutrient mixture F-12 (DMEM/F12) supplemented with 2% fetal bovine serum (Gibco, Grand Island, NY, USA), 100 U/mL penicillin (HyClone, Carlsbad, CA, USA), 0.1 mg/mL streptomycin (HyClone, Carlsbad, CA, USA), 15 μg/mL enrofloxacin (Aladdin, Shanghai, China), 0.1 mg/mL gentamicin, and 5 μg/mL amphotericin (Dingguo, Beijing, China), as well as additional epithelial cell growth factors (Cat No. CC-4175, Lonza, Basel, Switzerland).

### Transcriptional analysis of NOX gene expression levels of different *M. hyopneumoniae* strains

Five *M. hyopneumoniae* strains (strain 168, JS, LH, J and 168L) were cultured in KM2 cell-free liquid medium containing 20% (v/v) swine serum at 37 °C for 48 h, and the titer of these five *M. hyopneumoniae* strains were adjusted to 1 × 10^8^ CCU/mL before the next experiment. And then total RNA was extracted using a Total RNA Extraction Kit (Omega). No less than 1 μg of total RNA was reverse transcribed in a 10 µL reaction volume using HiScript® III RT SuperMix for qPCR (+gDNA wiper) (Cat No. R323-01, Vazyme, Nanjing, China) before running on an ABI 7500 Real Time PCR System using the ChamQ Universal SYBR qPCR Master Mix (Cat No. Q711-02, Vazyme, Nanjing, China). The reverse transcription reaction was followed the manufacturer’s protocol, and the qRT-PCR was performed using cDNA under specific conditions for the *nox* gene with the following procedure: samples were heated at 95 °C for 30 s, and a two-step cycle (10 s at 95 °C, 30 s at 60 °C) was repeated for 40 cycles. The *MHP7448_0333* gene of *M. hyopneumoniae* was used as the internal control [38, 39]. The PCR primers used in the quantitative assays are listed in Supplementary Table S1. The fold change of mRNA expression of *nox* gene between different *M. hyopneumoniae* strains was determined using the 2^-ΔΔCT^ method [40].

### Bioinformatic analysis, cloning, expression and purification of rNOX

Eighteen amino acid sequences of NOX from *M. hyopneumoniae* were retrieved from the National Center for Biotechnology Information (NCBI) and UniProt protein databases, and the homologies among them were analyzed. All sequences were aligned with CLUSTAL W program (open source). Molecular Evolutionary Genetics Analysis version 10 (MAGE10) was used for assessing phylogenetic inference according to the neighbor-joining criterion. The robustness of the hypothesis was evaluated with 1000 nonparametric bootstrap analyses.

The *M. hyopneumoniae nox* gene (MHP168_RS00400) was synthesized by GenScript Biotech Corp. (Nanjing, China) and then expressed using the pET-21a vector in the BL21(DE3) *E. coli* strain. The rNOX protein was purified using high affinity Ni-Charged resin and identified by Western blot analysis. Detailed protocols are provided in supplementary file 1 (S1).

### Preparation of polyclonal antibody recognizing rNOX

Two 1-month-old New Zealand white rabbits were immunized with rNOX protein. After three immunizations at 2-week intervals, sera were collected and purified using a HiTrap Protein G HP antibody purification column. Detailed protocols are provided in supplementary file 1 (S1).

### Enzymatic activity assays

The enzymatic activity of purified rNOX was determined by measuring the oxidation of NADH to NAD^+^ at a temperature of 25 °C as previously described with modifications [7, 41]. The reaction system contained 0.1 M potassium phosphate buffer (pH 7.5) and 1 mM dithiothreitol. The reaction system had a volume of 2 mL, purified rNOX was added at a final concentration of 5 μg/mL, and 10 μM flavin adenine dinucleotide (FAD) was added (Cat No. F100990, Aladdin, Shanghai, China). NADH (0.5 mM) Cat No. N106933, Aladdin, Shanghai, China) was added followed by incubation for 5 min at 25 °C. The optical density (OD) at 340 nm (OD_340_) was measured. The specific activity of rNOX was calculated as follows.

$$\mathrm{U}/\mathrm{mg}=\frac{\Delta \mathrm{OD}}{t\times \varepsilon \times l\times m}\times V\times {10}^{6}$$

### Detection of surface‑exposed NOX by flow cytometry

To investigate whether NOX is present on the surface of *M. hyopneumoniae* strains and to probe NOX surface distribution differences between highly virulent strain 168 and virulence-attenuated strain 168L, fluorescence intensity was measured using a flow cytometer as previously described with modifications [22]. Briefly, *M. hyopneumoniae* strains 168 and 168L (each 1 × 10^8^ CCU/mL) were incubated with anti-rNOX serum at a 1:100 dilution (1:100 diluted pre-immune serum was used as a negative control). *M. hyopneumoniae* strains were then stained with fluorescein isothiocyanate (FITC)-conjugated anti-IgG (Cat No. BA1105, Boster, Wuhan, China), and the fluorescence intensity was measured using a BD Accuri C6 flow cytometer.

### Subcellular localization of NOX in different virulent *M. hyopneumoniae* strains

Membrane proteins and cytoplasmic proteins of *M. hyopneumoniae* were obtained using a membrane protein and cytoplasmic protein extraction kit (Cat No. BB-3111, Bestbio, Shanghai, China) according to the manufacturer’s instructions. At the same time, the washed bacterial precipitate was resuspended in PBS and sonicated to prepare whole bacterial protein. Protein concentration was determined by the BCA Protein Assay Kit (Cat No. P0012S, Beyotime, Shanghai, China). Subcellular localization of NADH oxidase in various *M. hyopneumoniae* strains that differed in virulence was assessed by Western blot. Detailed protocols are provided in supplementary file 1 (S1).

### Adherence of rNOX to hTERT-PBECs

hTERT-PBECs were grown to confluence in 24-well plates with DMEM/F12 medium supplemented with 2% (v/v) fetal bovine serum plus the abovementioned growth factors. Cultured hTERT-PBECs were washed with PBS three times and fixed with 4% paraformaldehyde for 10 min at room temperature. Subsequently, the cells were treated with 0.1% Triton X-100 and then blocked for 2 h in 3% (w/v) BSA in PBS. After incubation with different concentrations of purified rNOX (from 5 to 500 μg), the cells were washed three times with PBS and incubated with a 1:250 dilution anti-rNOX antibody for 2 h at 37 °C. Following three PBS washes, cells were incubated with tetraethyl rhodamine isothiocyanate (TRITC)-tagged anti-IgG (Cat No. SA00007-2, Proteintech, Rosemont, IL, USA) at a 1:100 dilution for 1 h at 37 °C. Finally, nuclei were stained with DAPI (Cat No. D8417, Sigma-Aldrich, St Louis, MO, USA) before cells were observed using a fluorescence microscope (Zeiss). BSA and rGroEL (the cytoplasmic chaperone protein of *Aeromonas hydrophila*) [42] were used instead of rNOX as the negative control.

### Inhibition of adherence using antibody recognizing rNOX

*M. hyopneumoniae* bacteria (strains 168 and 168L, 1 × 10^7^ CCU/mL) were collected and washed three times with PBS before resuspension in 500 µL PBS. The samples were preincubated with polyclonal antibody raised against rNOX or pre-immune sera (1:20 dilution) at 37 °C for 30 min. hTERT-PBECs with a confluence of 85% in 24-well cell plates were washed with PBS three times before the experiment. Bacteria suspended in DMEM/F12 medium were added to cells seeded in 24-well cell plates, and the cell plates were centrifuged at 800 × *g* for 10 min and then incubated for 2 h at 4 °C. After washing with PBS three times, the cells in each group were treated with lysis buffer containing 0.1% trypsin. Bacterial counting, including bacterial genome extraction and real-time PCR, was performed according to a previous method [43]. The real-time PCR primers used in the quantitative assays are listed in Supplementary Table S1. In addition, the titers of different *M. hyopneumoniae* were quantified using a CCU_50_ assay as mentioned above. These tests were performed in triplicate, and data were analyzed using GraphPad Prism 6 and SPSS 20.0.

### Identification of rNOX-binding ligands

As previously described with some modifications [7], the recombinant protein rNOX and the negative control proteins rGroEL were serially diluted twofold from 2 μg/ml to 0.5 μg/mL, and PBS was used as a blank control rather than rNOX. For each dilution, 100 μL per well was coated (in triplicate) in a 96-well plate. The wells were blocked with 5% BSA (w/v) in PBS containing 0.5% Tween 20 (PBST) for 2 h at 37 °C. Two hundred micrograms of fibronectin (Cat No. F1056, Sigma–Aldrich, St Louis, MO, USA) in 100 μL of PBST was applied to the wells, and ELISA plates were incubated at 37 °C for 1 h. Wells were washed three times with PBST and incubated with a 1:2000 dilution of anti-fibronectin antibody (Cat No. ab299, Abcam, Cambridge, UK) for 1 h at 37 °C. Following three washes with PBST, the wells were incubated with HRP-conjugated goat anti-rabbit IgG antibody (Cat No. BA1055, Boster, Wuhan, China, 1:5000 in PBST) at 37 °C for 1 h. Finally, a 3,3',5,5'-Tetramethylbenzidine (TMB) (Cat No. P0209, Beyotime, Shanghai, China) substrate was applied at 37 °C, and the wells were treated with the stop solution (2 M H_2_SO_4_). OD readings were obtained at a wavelength of 450 nm with an ELISA microplate reader (Bio-Tek, Winooski, VT, USA).

Each 20 μg sample of rNOX was resolved by 10% SDS-PAGE and then transferred to a PVDF membrane (Cat No. IPFL00010, Millipore, Darmstadt, Germany). The membranes were washed three times with PBS and then blocked with 5% skimmed milk in TBST (TBS containing 0.5% Tween 20) at 37 °C for 2 h. Subsequently, the membranes were incubated with 15 μg/mL fibronectin (Cat No. F1056, Sigma-Aldrich, St Louis, MO, USA) for 2 h at 37 °C. Following three washes using TBST, the membranes were incubated with anti-fibronectin antibody (Cat No. ab299, Abcam, Cambridge, UK, 1:1000 dilution) in blocking solution at 37 °C for 2 h, after which they were washed three times with TBST followed by incubation with horseradish peroxidase (HRP)-conjugated goat anti-rabbit IgG (Cat No. BA1055, Boster, Wuhan, China) at 37 °C for 2 h. Finally, the membranes were developed with Electro-Chemi-Luminescence (ECL, Cat No. 32109, ThermoFisher, Rockford, IL, USA) substrate using a ChemiDoc XRS+ system (Bio–Rad). BSA was used as a negative control rather than rNOX.

### Quantification of lactate dehydrogenase (LDH) Release

hTERT-PBECs were incubated for 6 h and grown to confluence in 24-well plates. After three washes with PBS, the cells were then incubated with purified rNOX protein at different concentrations (5 μg, 10 μg, 15 μg, and 20 μg). PBS was used as a negative control rather than rNOX, and LPS (5 μg/mL), DMSO (1% diluted in DMEM/F12 medium), and four *M. hyopneumoniae* strains (strains JS, 168, J and 168L, titers of which were 1 × 10^8^ CCU/mL) were used as positive controls. All groups were performed with three independent replicates. After 6 h, culture supernatants were collected, and LDH activity was measured using a CytoTox 96^®^ Non-Radioactive Cytotoxicity Assay (Cat No. G1780, Promega, Madison, WI, USA) according to the manufacturer’s instructions.

### ROS detection

hTERT-PBECs were seeded and grown to confluence in 24-well plates. The cells were washed with PBS followed by incubation with purified rNOX protein of different concentrations (from 5 to 20 μg) at 37 °C for 6 h. At the same time, PBS was used as a negative control, and cells treated with LPS (5 μg/mL), DMSO (1%), and four *M. hyopneumoniae* strains (strain JS, 168, J and 168L, 1 × 10^8^ CCU/mL) were used as positive controls. All tests were performed with five independent replicates. Reactive oxygen species (ROS) were measured using a ROS-Glo™ H_2_O_2_ Assay (Cat No. G8820, Promega, Madison, WI, USA) according to the manufacturer’s instructions, and RLUs were recorded using a plate reader.

### Induced apoptosis in hTERT-PBECs measured by flow cytometry

hTERT-PBECs were seeded in 24-well plates at 2×10^5^ cells/well in 500 µL of culture medium. Then, hTERT-PTECs were incubated with 20 μg purified rNOX at 37 °C for 12 h. Cells cultured in DMEM/F12 plus 2% FBS culture medium supplemented with epithelial growth factors F12 without the reagents mentioned above were used as a negative control, and cells incubated with *M. hyopneumoniae* strains (strains 168 and 168L, 1 × 10^8^ CCU/mL) were used as positive controls. At the same time, to explore whether antiserum to rNOX, which could block the apoptosis induced by *M. hyopneumoniae* in hTERT-PBECs, we pre-incubated *M. hyopneumoniae* strains (168 and 168L) with anti-rNOX antibody (1:20 dilution) at 37 °C for 30 min before inoculation with hTERT-PBEC seeds in 24-well plates at 37 °C for 12 h. All groups were performed with three independent replicates. Apoptosis rates were detected with the dual staining Annexin V- fluorescein isothiocyanate / propidium iodide (Annexin V- FITC/PI) method using an Annexin V-FITC/PI Apoptosis Detection Kit (Cat No. A211, Vazyme, Nanjing, China).

### Statistical analysis

All experiments were repeated in triplicate, except for the detection of ROS, which was conducted in five independent replicates. Data represent the mean ± standard deviation (SD) of three or five individual experiments. Relative mRNA expression differences of the *nox* gene between the virulence-attenuated strain 168L and the virulent Mhp strains 168, JS, LH and J were assessed via one-way analysis of variance (ANOVA) using SPSS Statics v20.0 software and GraphPad Prism 6 software. The adhesion rates between anti-NOX serum or negative serum and groups of the high-virulence strain 168 as well as the adhesion rates between the low-virulence strain 168L and anti-NOX serum or negative serum were compared by Student’s *t* test. *P* < 0.05 was considered statistically significant, *P* < 0.01 was considered highly significant, and *P* < 0.001 was considered extremely significant.

## Supplementary Information

**Additional file 1: S1.** **Table S1.** Primers used in this study. **Fig. S1.** (A) Multiple sequence alignment of proteins NOX from different *M. hyopneumoniae *strains. (B) Phylogenetic analysis based on the NOX proteins of different *M. hyopneumoniae* strains by the neighbor-joining (NJ) method with the sequences available in GenBank and Uniprot Database. The numbers at the phylogenetic branches indicated the bootstrap values (1000 replicates) in percentage supporting each group. The bar represents the genetic distance. **Fig. S2.** Prokaryotic expression and purification of recombinant protein, WB verification of prepared polyclonal antibody. “a”, Lane M is prestained protein mass markers, with recombinant protein rNOX size of 50.6 KDa. The prepared polyclonal antibody effect of the recombinant protein rNOX was tested by Western Blot. “b”, a clear band appeared at position of 50.6 KDa. Lane 1, purified recombinant protein rNOX. Lane 2, unpurified *E. coli* whole bacteria protein before purification. Lane M, prestained protein mass marker. **Fig. S3.** SDS–PAGE analysis of purified recombinant protein of rNOX, other proteins of *M. hyopneumoniae* (Mhp) and BSA. Lane M, prestained protein mass marker. Lane 1, purified recombinant protein rNOX. Lane 2, bovine serum albumin (BSA). Lane 3, purified recombinant nicotinamide adenine dinucleotide-dependent flavin oxidoreductase (NFOR) protein of Mhp. Lane 4 and 6, blank. Lane 5 and 7, purified recombinant leucine aminopeptidase (LAP) protein of Mhp. **Fig. S4.** *M. hyopneumoniae* proteins with fibronectin interaction analysis by far Western blot. Lane M, prestained protein mass marker. Lane 1, purified recombinant protein rNFOR. PVDF membrane with transferred Mhp NFOR protein incubated with fibronectin and the anti-fibronectin antibody. Lane 2, 4 and 8, BSA, PVDF membrane with transferred BSA (negative control) incubated with fibronectin and the anti-fibronectin antibody. Lane 3, purified recombinant protein rNOX. PVDF membrane with transferred Mhp NOX protein incubated with fibronectin and the anti-fibronectin antibody. Lane 5, 6, 7 and 9, purified recombinant protein rLAP. PVDF membrane with transferred Mhp LAP protein incubated with fibronectin and the anti-fibronectin antibody.

## Data Availability

All data generated or analysed during this study are included in this published article and its supplementary information files.

## Abbreviations

ANOVA: analysis of variance
bp: base pairs
BSA: bovine serum albumin
CCU: color changing unit
CCU_50_: 50% color changing unit
DAPI: 6-diamidino-2-phenylindole
DMEM/F12: Dulbecco's modified Eagle’s medium nutrient mixture F-12
DMSO: dimethyl sulfoxide
ECL: Electro-Chemi-Luminescence
EP: Enzootic pneumonia
FAD: flavin adenine dinucleotide
FITC: fluorescein isothiocyanate
GAPDH: Glyceraldehyde-3-phosphate dehydrogenase
GroEL: heat shock protein Hsp60
HRP: horseradish peroxidase
hTERT-PBECs: immortalized porcine bronchial epithelial cells
LDH: lactate dehydrogenase
LPS: lipopolysaccharides
IFA: Indirect immunofluorescence assay
ISMPs: intracellular enzymes or intracellular / surface moonlighting proteins
MAGE10: Molecular Evolutionary Genetics Analysis version 10
MFI: the mean fluorescence intensity
Mhp: *Mycoplasma hyopneumoniae*
NADH: nicotinamide adenine dinucleotide
NCBI: National Center for Biotechnology Information
NFOR: NADH-dependent flavin oxidoreductase
NOX: NADH oxidase
OD: optical density
PBS: phosphate buffered saline
PBST: phosphate buffered saline containing 0.5% Tween 20
PCV: porcine circovirus
PI: propidium iodide
PRDC: porcine respiratory disease complex
PRRSV: porcine reproductive and respiratory syndrome virus
qPCR: quantitative polymerase chain reaction
rNOX: recombinant NOX protein
RLUs: relative luminescence units
ROS: reactive oxygen species
RT-PCR: reverse transcription polymerase chain reaction
SD: standard deviation
sF-pCD: snatch-farrowed, porcine-colostrum-deprived
TMB: 3,3',5,5'-Tetramethylbenzidine
TRITC: tetraethyl rhodamine isothiocyanate

## References

[1] Opriessnig T, Gimenez-Lirola LG, Halbur PG (2011). Polymicrobial respiratory disease in pigs. Anim Health Res Rev..

[2] Maes D, Sibila M, Kuhnert P, Segales J, Haesebrouck F, Pieters M (2018). Update on *Mycoplasma hyopneumoniae* infections in pigs: Knowledge gaps for improved disease control. Transbound Emerg Dis..

[3] Thacker EL, Thacker BJ, Janke BH (2001). Interaction between *Mycoplasma hyopneumoniae* and swine influenza virus. J Clin Microbiol..

[4] Saade G, Deblanc C, Bougon J, Marois-Crehan C, Fablet C, Auray G, Belloc C, Leblanc-Maridor M, Gagnon CA, Zhu J (2020). Coinfections and their molecular consequences in the porcine respiratory tract. Vet Res..

[5] Leal Zimmer FMA, Paes JA, Zaha A, Ferreira HB (2020). Pathogenicity & virulence of *Mycoplasma hyopneumoniae*. Virulence..

[6] Rohmer L, Hocquet D, Miller SI (2011). Are pathogenic bacteria just looking for food? Metabolism and microbial pathogenesis. Trends Microbiol..

[7] Zhao G, Zhang H, Chen X, Zhu X, Guo Y, He C, Anwar Khan F, Chen Y, Hu C, Chen H (2017). *Mycoplasma bovis* NADH oxidase functions as both a NADH oxidizing and O_2_ reducing enzyme and an adhesin. Sci Rep..

[8] Tacchi JL, Raymond BB, Haynes PA, Berry IJ, Widjaja M, Bogema DR, Woolley LK, Jenkins C, Minion FC, Padula MP (2016). Post-translational processing targets functionally diverse proteins in *Mycoplasma hyopneumoniae*. Open Biol..

[9] Berry IJ, Jarocki VM, Tacchi JL, Raymond BBA, Widjaja M, Padula MP, Djordjevic SP (2017). N-terminomics identifies widespread endoproteolysis and novel methionine excision in a genome-reduced bacterial pathogen. Sci Rep..

[10] Paes JA, Machado L, Dos Anjos Leal FM, De Moraes SN, Moura H, Barr JR, Ferreira HB (2018). Comparative proteomics of two *Mycoplasma hyopneumoniae* strains and *Mycoplasma flocculare* identified potential porcine enzootic pneumonia determinants. Virulence..

[11] Pinto PM, Klein CS, Zaha A, Ferreira HB (2009). Comparative proteomic analysis of pathogenic and non-pathogenic strains from the swine pathogen *Mycoplasma hyopneumoniae*. Proteome Sci..

[12] Zheng C, Ren S, Xu J, Zhao X, Shi G, Wu J, Li J, Chen H, Bei W (2017). Contribution of NADH oxidase to oxidative stress tolerance and virulence of *Streptococcus suis* serotype 2. Virulence..

[13] Raymond BBA, Madhkoor R, Schleicher I, Uphoff CC, Turnbull L, Whitchurch CB, Rohde M, Padula MP, Djordjevic SP (2018). Extracellular actin is a receptor for *Mycoplasma hyopneumoniae*. Front Cell Infect Microbiol..

[14] Jeffery C (2018). Intracellular proteins moonlighting as bacterial adhesion factors. AIMS Microbiol..

[15] Peterson SN, Fraser CM (2001). The complexity of simplicity. Genome Biol..

[16] Razin S, Yogev D, Naot Y (1998). Molecular biology and pathogenicity of mycoplasmas. Microbiol Mol Biol Rev..

[17] Raymond BBA, Jenkins C, Turnbull L, Whitchurch CB, Djordjevic SP (2018). Extracellular DNA release from the genome-reduced pathogen *Mycoplasma hyopneumoniae* is essential for biofilm formation on abiotic surfaces. Sci Rep..

[18] Raymond BBA, Turnbull L, Jenkins C, Madhkoor R, Schleicher I, Uphoff CC, Whitchurch CB, Rohde M, Djordjevic SP (2018). *Mycoplasma hyopneumoniae* resides intracellularly within porcine epithelial cells. Sci Rep..

[19] Li G, Obeng E, Shu J, Shu J, Chen J, Wu Y, He Y (2020). Genomic variability and post-translational protein processing enhance the immune evasion of *Mycoplasma hyopneumoniae* and its interaction with the porcine immune system. Front Immunol..

[20] Antikainen J, Kuparinen V, Lahteenmaki K, Korhonen TK (2007). pH-dependent association of enolase and glyceraldehyde-3-phosphate dehydrogenase of *Lactobacillus crispatus* with the cell wall and lipoteichoic acids. J Bacteriol..

[21] Daubenspeck JM, Liu R, Dybvig K (2016). Rhamnose links moonlighting proteins to membrane phospholipid in Mycoplasmas. PLoS One..

[22] Xie X, Hao F, Chen R, Wang J, Wei Y, Liu J, Wang H, Zhang Z, Bai Y, Shao G (2021). Nicotinamide adenine dinucleotide-dependent flavin oxidoreductase of *Mycoplasma hyopneumoniae* functions as a potential novel virulence factor and not only as a metabolic enzyme. Front Microbiol..

[23] Blanchard B, Vena MM, Cavalier A, Le Lannic J, Gouranton J, Kobisch M (1992). Electron microscopic observation of the respiratory tract of SPF piglets inoculated with *Mycoplasma hyopneumoniae*. Vet Microbiol..

[24] Xie X, Gan Y, Pang M, Shao G, Zhang L, Liu B, Xu Q, Wang H, Feng Y, Yu Y (2018). Establishment and characterization of a telomerase-immortalized porcine bronchial epithelial cell line. J Cell Physiol..

[25] Henderson B, Nair S, Pallas J, Williams MA (2011). Fibronectin: a multidomain host adhesin targeted by bacterial fibronectin-binding proteins. FEMS Microbiol Rev..

[26] Seymour LM, Deutscher AT, Jenkins C, Kuit TA, Falconer L, Minion FC, Crossett B, Padula M, Dixon NE, Djordjevic SP (2010). A processed multidomain *mycoplasma hyopneumoniae* adhesin binds fibronectin, plasminogen, and swine respiratory cilia. J Biol Chem..

[27] Jing X, Park JH, Peters TM, Thorne PS (2015). Toxicity of copper oxide nanoparticles in lung epithelial cells exposed at the air-liquid interface compared with in vivo assessment. Toxicol In Vitro..

[28] Bai F, Ni B, Liu M, Feng Z, Xiong Q, Xiao S, Shao G (2013). *Mycoplasma hyopneumoniae*-derived lipid-associated membrane proteins induce apoptosis in porcine alveolar macrophage via increasing nitric oxide production, oxidative stress, and caspase-3 activation. Vet Immunol Immunopathol..

[29] Grant SS, Hung DT (2013). Persistent bacterial infections, antibiotic tolerance, and the oxidative stress response. Virulence..

[30] Obeng E (2021). Apoptosis (programmed cell death) and its signals - A review. Braz J Biol..

[31] Simon HU, Haj-Yehia A, Levi-Schaffer F (2000). Role of reactive oxygen species (ROS) in apoptosis induction. Apoptosis..

[32] Li Y, Jiang Z, Xue D, Deng G, Li M, Liu X, Wang Y (2016). *Mycoplasma ovipneumoniae* induces sheep airway epithelial cell apoptosis through an ERK signalling-mediated mitochondria pathway. BMC Microbiol..

[33] Ho C, Chu T, Chin H, Mao H, Yeh A, Chen C, Chang S, Chang D (1980). Microagglutination test for the diagnosis of swine *mycoplasmal pneumonia* and the identification of Mycoplasmas. Acta Vet Zootech Sinica..

[34] Liu W, Xiao S, Li M, Guo S, Li S, Luo R, Feng Z, Li B, Zhou Z, Shao G (2013). Comparative genomic analyses of *Mycoplasma hyopneumoniae* pathogenic 168 strain and its high-passaged attenuated strain. BMC Genomics..

[35] Xiong Q, Wei Y, Feng Z, Gan Y, Liu Z, Liu M, Bai F, Shao G (2014). Protective efficacy of a live attenuated *Mycoplasma hyopneumoniae* vaccine with an ISCOM-matrix adjuvant in pigs. Vet J..

[36] Leigh SA, Evans JD, Branton SL, Collier SD (2008). The effects of increasing sodium chloride concentration on *Mycoplasma gallisepticum* vaccine survival in solution. Avian Dis..

[37] Furr PM, Taylor-Robinson D (1993). Factors influencing the ability of different mycoplasmas to colonize the genital tract of hormone-treated female mice. Int J Exp Pathol..

[38] Moitinho-Silva L, Heineck BL, Reolon LA, Paes JA, Klein CS, Rebelatto R, Schrank IS, Zaha A, Ferreira HB (2012). *Mycoplasma hyopneumoniae* type I signal peptidase: expression and evaluation of its diagnostic potential. Vet Microbiol..

[39] Merker Breyer G, Malvessi Cattani A, Silveira Schrank I, Maboni Siqueira F (2022). The influence of regulatory elements on *Mycoplasma hyopneumoniae* 7448 transcriptional response during oxidative stress and heat shock. Mol Biol Rep..

[40] Livak KJ, Schmittgen TD (2001). Analysis of relative gene expression data using real-time quantitative PCR and the 2(T)(-Delta Delta C) method. Methods..

[41] Nisar MA, Rashid N, Bashir Q, Gardner QT, Shafiq MH, Akhtar M (2013). TK1299, a highly thermostable NAD(P)H oxidase from Thermococcus kodakaraensis exhibiting higher enzymatic activity with NADPH. J Biosci Bioeng..

[42] Liu J, Dong Y, Wang N, Ma S, Lu C, Liu Y (2019). Diverse effects of nitric oxide reductase NorV on Aeromonas hydrophila virulence-associated traits under aerobic and anaerobic conditions. Vet Res..

[43] Wu Y, Jin M, Bai F, Zhang X, Hua L, Lei Z, Shao G (2012). Development and application of TaqMan-BHQ real time PCR assay for detection of *Mycoplasma hyopneumoniae* P97. Chin Vet Sci..

