# Supplementary material for: NADH oxidase of Mycoplasma hyopneumoniae functions as a potential mediator of virulence
Source: BMC Vet Res. 2022 Apr 2;18:126. doi: 10.1186/s12917-022-03230-7 (PMC8976378; doi:10.1186/s12917-022-03230-7)
Supplement: Supplementary file 1 — Additional file 1: S1. Table S1. Primers used in this study. Fig. S1. (A) Multiple sequence alignment of proteins NOX from different M. hyopneumoniae strains. (B) Phylogenetic analysis based on the NOX proteins of different M. hyopneumoniae strains by the neighbor-joining (NJ) method with the sequences available in GenBank and Uniprot Database. The numbers at the phylogenetic branches indicated the bootstrap values (1000 replicates) in percentage supporting each group. The bar represents the genetic distance. Fig. S2. Prokaryotic expression and purification of recombinant protein, WB verification of prepared polyclonal antibody. “a”, Lane M is prestained protein mass markers, with recombinant protein rNOX size of 50.6 KDa. The prepared polyclonal antibody effect of the recombinant protein rNOX was tested by Western Blot. “b”, a clear band appeared at position of 50.6 KDa. Lane 1, purified recombinant protein rNOX. Lane 2, unpurified E. coli whole bacteria protein before purification. Lane M, prestained protein mass marker. Fig. S3. SDS–PAGE analysis of purified recombinant protein of rNOX, other proteins of M. hyopneumoniae (Mhp) and BSA. Lane M, prestained protein mass marker. Lane 1, purified recombinant protein rNOX. Lane 2, bovine serum albumin (BSA). Lane 3, purified recombinant nicotinamide adenine dinucleotide-dependent flavin oxidoreductase (NFOR) protein of Mhp. Lane 4 and 6, blank. Lane 5 and 7, purified recombinant leucine aminopeptidase (LAP) protein of Mhp. Fig. S4. M. hyopneumoniae proteins with fibronectin interaction analysis by far Western blot. Lane M, prestained protein mass marker. Lane 1, purified recombinant protein rNFOR. PVDF membrane with transferred Mhp NFOR protein incubated with fibronectin and the anti-fibronectin antibody. Lane 2, 4 and 8, BSA, PVDF membrane with transferred BSA (negative control) incubated with fibronectin and the anti-fibronectin antibody. Lane 3, purified recombinant protein rNOX. PVDF membrane with transferred Mhp N [file 12917_2022_3230_MOESM1_ESM.docx]

**Supplementary file 1 (S1)**

**Bioinformatic analysis, Cloning, expression and purification of recombinant NOX (rNOX)**

Eighteen amino acid sequences of NOX from *M. hyopneumoniae* were retrieved from the National Center for Biotechnology Information and UniProt protein database and the homologies among them were analyzed. All sequences were aligned with CLUSTAL W program [[1](#_ENREF_1)]. Molecular Evolutionary Genetics Analysis version 10 (MAGE10) [[2](#_ENREF_2)] was used for phylogeny inference according to the neighbor-joining criterion. The robustness of the hypothesis was test with 1000 nonparametric bootstrap analyses. The accession number of the eighteen amino acid sequences are listed as follows: WP 129640626.1, VEU66106.1, AGM21864.1, WP 016340184.1, ADQ90307.2, WP 014579578.1, OWG15588.1, WP 011206136.1, AAV27813.1, QBY87409.1, WP 135618531.1, WP 011283895.1, ASU14227.1, QEA02294.1, AAZ44172.1, AAZ53459.1, AGQ50697.1, and WP 011289994.1.

The *M. hyopneumoniae nox* gene (MHP168_RS00400) was synthesized by GenScript Biotech Corp. (Nanjing, China), expressed using the pET-21a vector in the BL21(DE3) *E. coli* strain. Before protein expression, NOX was analyzed for signal peptide predictions with SignalP-5.0 Server1 [[3](#_ENREF_3)]. The protein expression, purification and renaturation processes were performed as previously reported with some modifications [[4](#_ENREF_4), [5](#_ENREF_5)]. In detail, bacterial cells were grown in LB medium at 37 °C until the OD600 reached approximately 0.7, and then, IPTG was added to the culture at a final concentration of 1 mM and incubated at 37 °C and 180 rpm for 5 h to induce protein expression. The cells were harvested by centrifugation and resuspended in lysis buffer (containing 30 mM Tris-HCl (pH 8.0), 300 mM NaCl, 20 mM imidazole and 2% Glycerol) before lysed by sonication. Intracellular compounds were isolated by 15 000 × *g* centrifugation at 4 °C for 15 min. Cell pellets were washed three times in wash buffer (20 mM Na_3_PO_4_, 0.5 M NaCl, 30 mM imidazole, and 2 M urea, pH 8.0), then resuspended in binding buffer (8 M urea, 20 mM Na_3_PO_4_, 0.5 M NaCl, 30 mM imidazole, 1 mM β-mercaptoethanol in PBS, pH 8.0) and was allowed to dissolve overnight at 4 °C. After centrifugation at 15 000× *g* for 30 min to remove any insoluble debris, the supernatant was applied on High Affinity Ni-Charged Resin FF affinity columns (Cat No. L00666, Genscript, Nanjing, China) equilibrated with binding buffer. The column was washed with 100 mL binding buffer followed by 20 ml elution buffer (buffer A supplemented with 500 mM imidazole).

Subquently, the purified denaturation recombinant protein was stepwise dialyzed at 4 °C with the following refolding buffers: (a) refolding buffer A (4 M urea, 0.1 mM glutathione, 0.01 mM glutathione disulfide, 1 mM EDTA, 5% (v/v) glycerol in PBS, pH = 8.0) for 12 h; (b) refolding buffer B (2 Murea, 0.1 mMglutathione, 0.01 mMglutathione disulfide, 1 mM EDTA, 5% (v/v) glycerol, 0.15 M L-arginine in PBS, pH = 8.0) for 12 h; (c) refolding buffer C (1 M urea, 0.1 mM glutathione, 0.01 mM glutathione disulfide, 1 mM EDTA, 5% (v/v) glycerol, in PBS, pH = 8.0) for 12 h; and (4) refolding buffer D (PBS, pH = 8.0) for 12 h, twice. Subsequently, the renatured protein was concentrated using Centricons (Cat No. UFC901024, Amicon, Merck Ltd., Beijing, China) ultrafiltration. The concentration of the purified proteins was determined with the BCA Protein Assay Kit (Cat No. P0012S, Beyotime, Shanghai, China) and identified by Western blot analysis. The proteins were aliquoted before stored at -70 °C.

**Preparation of polyclonal antibody recognizing rNOX**

The polyclonal antibody was raised against *M. hyopneumoniae* NOX by subcutaneously immunizing 1-month-old New Zealand white rabbits. All rabbits were immunized with 1 mg of rNOX emulsified in Freund's complete adjuvant for the first immunzation (Cat No. F5881, Sigma-Aldrich, St Louis, MO, USA). And each rabbit was immunized three times with 1 mg of rNOX emulsified in Freund's incomplete adjuvant (Cat No. F5506, Sigma-Aldrich, St Louis, MO, USA) at 2-week intervals. Sera were collected at 1 week after the third immunization. The antibody titers were measured by ELISA. Briefly, the 96-well plates were coated with purified rNOX (80 ng/well) at 4 °C overnight. After being washed three times with PBS containing 0.5% Tween 20 (PBST), unoccupied sites were blocked with 5% BSA in PBST for 2 h at 37 °C. The two-fold serial dilution of rabbit serum anti-NOX and preimmune serum were added to wells. Then, bound antibodies were detected by incubation with horseradish peroxidase (HRP)-conjugated goat anti-rabbit IgG (diluted 1:10 000, Cat No. BA1055, Boster, Wuhan, China) for 1 h at 37 °C. The antibody titer to rNOX was 1:204 800 (positive serum OD / negative serum OD ≥ 2.1). The polyclonal antibodies against rNOX were purified using a HiTrap Protein G HP antibody purification column (Cat No. 17040401, GE Healthcare, Boston, MA, USA).

**Subcellular localization of the NADH oxidase in different virulent** ***M. hyopneumoniae***

*M. hyopneumoniae* strains were grown to mid-log phase and harvested by centrifugation at 10 000 × *g* at 10 ℃ for 20 min and washed three times with PBS. The membrane proteins and cytoplasmic proteins of *M. hyopneumoniae* (strains 168 and 168L, 1 × 10^8^ CCU/mL) were obtained using a membrane protein and cytoplasmic protein extraction kit (Cat No. BB-3111, Bestbio, Shanghai, China) according to the manufacturer’s instructions. At the same time, the washed bacterial precipitate was resuspended with PBS and broken by sonication to prepare whole bacterial protein. The proteins concentration was determined by the BCA Protein Assay Kit (Cat No. P0012S, Beyotime, Shanghai, China). The protein samples were stored at -80°C until further use. 5 μg of membrane proteins and cytoplasmic proteins and whole bacterial proteins of *M. hyopneumoniae* strain 168 and 168L were resolved by 10% SDS-PAGE before being transferred to a PVDF membrane (Cat No. IPFL00010, Millipore, Darmstadt, Germany), respectively. The membranes were washed three times with PBS and then blocked with 5% skimmed milk in TBS containing 0.5% Tween 20 (TBST) at 37°C for 2 h with shaking. Subsequently, the membrane was incubated with anti-rNOX antibody (1:1000 dilution) in blocking solution at 37°C for 2 h. After washed three times with TBST, the membrane was incubated with the secondary antibody (HRP-conjugated goat anti-rabbit IgG) (Cat No. BA1055, Boster, Wuhan, China) at 37°C for 1 h. Finally, the membrane was developed with Electro-Chemi-Luminescence (ECL, Cat No. 32109, ThermoFisher, Rockford, IL, USA) substrate using a ChemiDoc XRS+ system (Bio-Rad).

**References**

1. Larkin MA, Blackshields G, Brown NP, Chenna R, McGettigan PA, McWilliam H, Valentin F, Wallace IM, Wilm A, Lopez R et al. Clustal W and Clustal X version 2.0. Bioinformatics. 2007;23(21):2947-8.

2. Kumar S, Stecher G, Li M, Knyaz C, Tamura K. MEGA X: Molecular Evolutionary Genetics Analysis across Computing Platforms. Mol Biol Evol. 2018;35(6):1547-9.

3. Almagro Armenteros JJ, Tsirigos KD, Sonderby CK, Petersen TN, Winther O, Brunak S, von Heijne G, Nielsen H. SignalP 5.0 improves signal peptide predictions using deep neural networks. Nat Biotechnol. 2019;37(4):420-3.

4. Shi T, Zhang L, Li Z, Newton IP, Zhang Q. Expression, purification and renaturation of truncated human integrin beta1 from inclusion bodies of *Escherichia coli*. Protein Expr Purif. 2015;107:13-9.

5. Milhim M, Gerber A, Neunzig J, Hannemann F, Bernhardt R. A Novel NADPH-dependent flavoprotein reductase from Bacillus megaterium acts as an efficient cytochrome P450 reductase. J Biotechnol. 2016;231:83-94.

**Supplementary tables and figures**

Table S1 Primers used in this study.

| Primers name | Summary of functions | Primer sequences (5’-3’) |
| --- | --- | --- |
| NOX-F | For real-time PCR analysis of *nox* gene expression at the transcriptional level | ATGCCGCAGACCTTGTGAT |
| NOX-R |  | AAGCAGCAACTATTCCTGATTT |
| Mhp0333-F |  | TGGGCAATCAAGAAGCAAC |
| Mhp0333-R |  | TGAAAACGGAAAACACCTTG |
| Mhp183-F | For real-time PCR analysis of *M. hyopneumoniae* | CCAGAACCAAATTCCTTCGCTG |
| Mhp183-R |  | ACTGGCTGAACTTCATCTGGGCTA |
| Mhp183-P |  | FAM-AGCAGATCTTAGTCAAAGTGCCCGTG-TAMRA |

Mhp means *M.hyopneumoniae.*

**Figure S1**

**
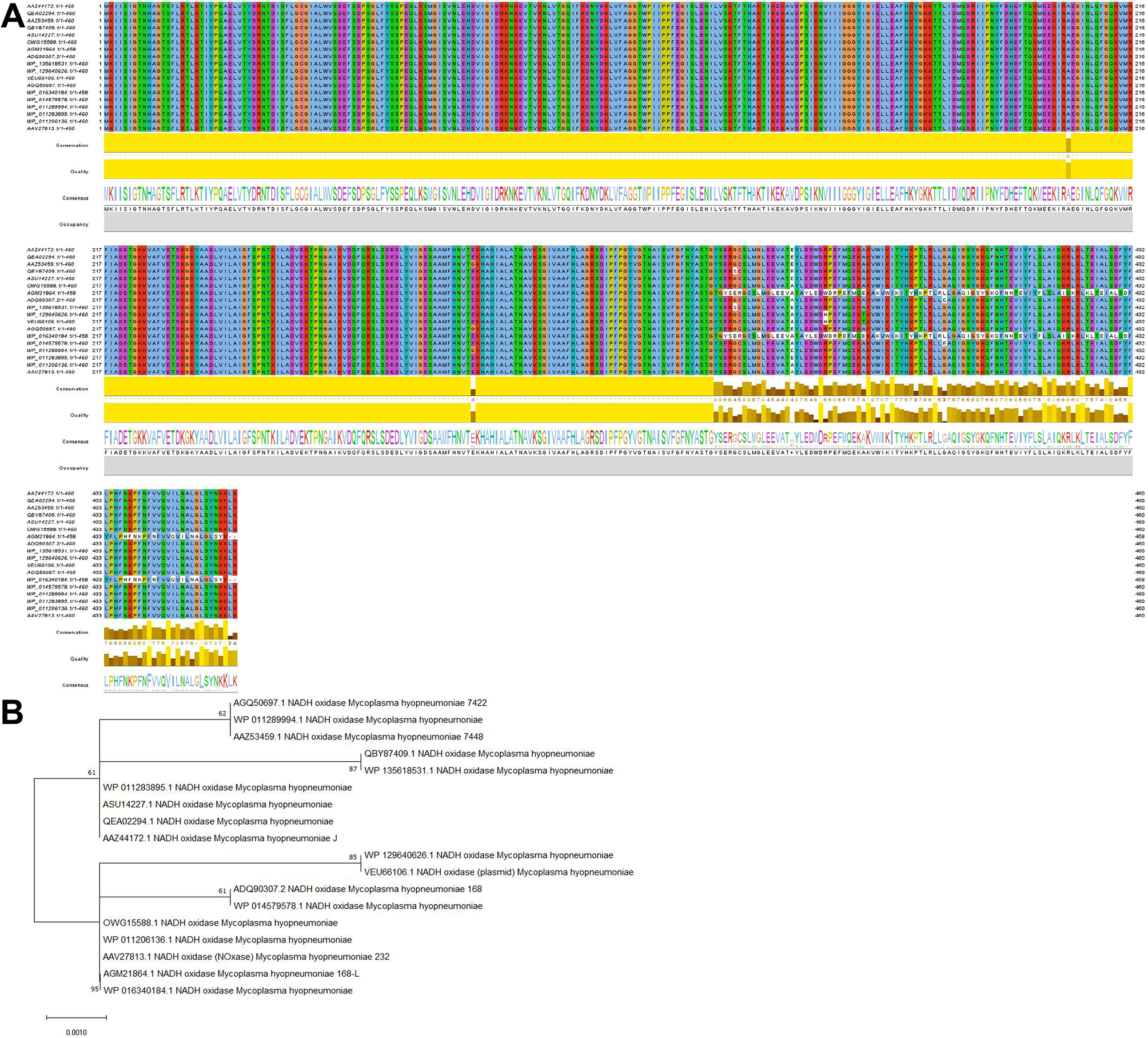
**

**Fig. S1.** (A) Multiple sequence alignment of proteins NOX from different *M. hyopneumoniae* strains. (B) Phylogenetic analysis based on the NOX proteins of different *M. hyopneumoniae* strains by the neighbor-joining (NJ) method with the sequences available in GenBank and Uniprot Database. The numbers at the phylogenetic branches indicated the bootstrap values (1000 replicates) in percentage supporting each group. The bar represents the genetic distance.

**Figure S2**

**
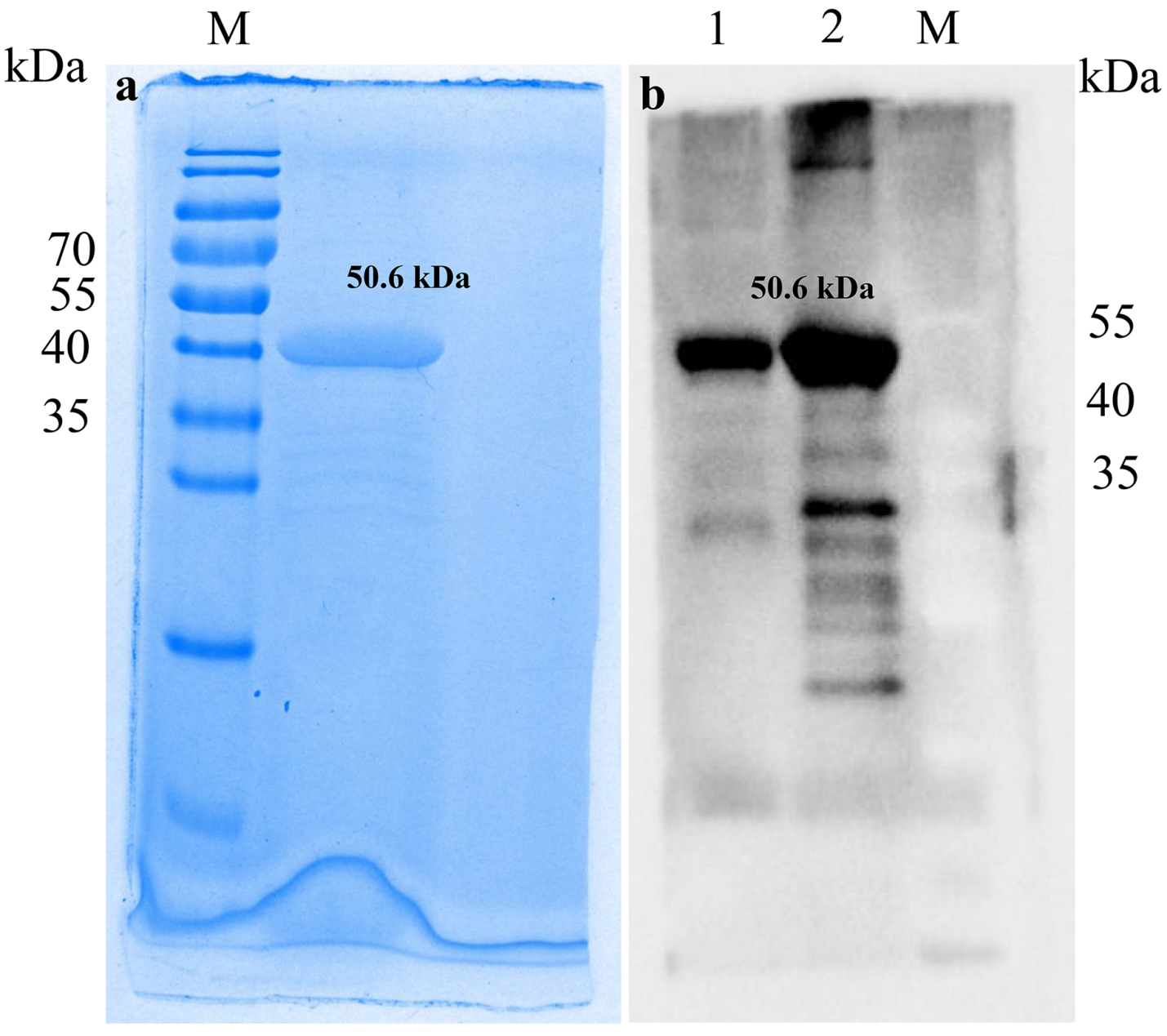
**

**Fig. S2.** Prokaryotic expression and purification of recombinant protein, WB verification of prepared polyclonal antibody. “a”, Lane M is prestained protein mass markers, with recombinant protein rNOX size of 50.6 KDa. The prepared polyclonal antibody effect of the recombinant protein rNOX was tested by Western Blot. “b”, a clear band appeared at position of 50.6 KDa. Lane 1, purified recombinant protein rNOX. Lane 2, unpurified *E. coli* whole bacteria protein before purification. Lane M, prestained protein mass marker.

**Figure S3**

**
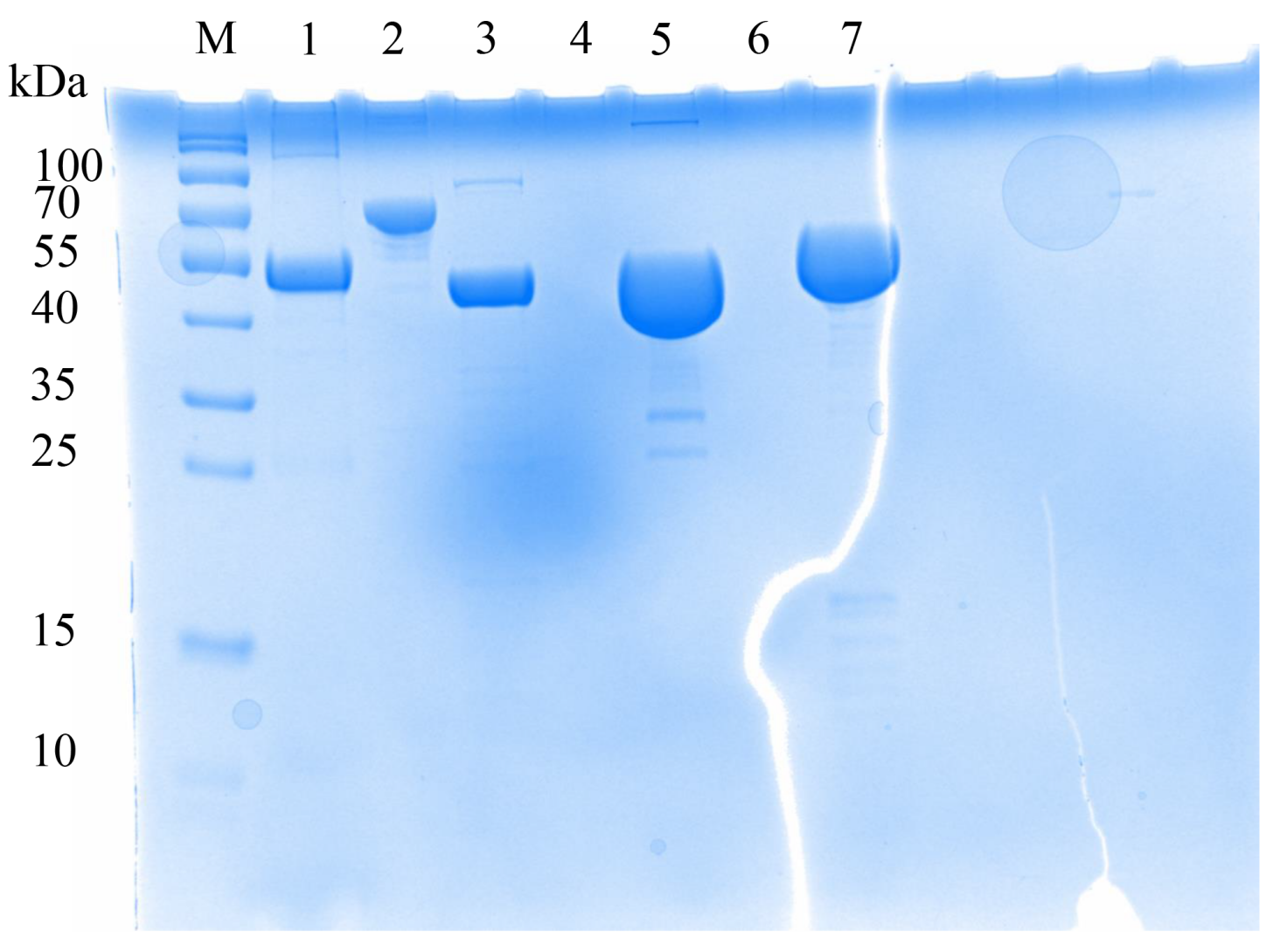
**

**Fig. S3.** SDS–PAGE analysis of purified recombinant protein of rNOX, other proteins of *M. hyopneumoniae* (Mhp) and BSA. Lane M, prestained protein mass marker. Lane 1, purified recombinant protein rNOX. Lane 2, bovine serum albumin (BSA). Lane 3, purified recombinant nicotinamide adenine dinucleotide-dependent flavin oxidoreductase (NFOR) protein of Mhp. Lane 4 and 6, blank. Lane 5 and 7, purified recombinant leucine aminopeptidase (LAP) protein of Mhp.

**Figure S4**

**
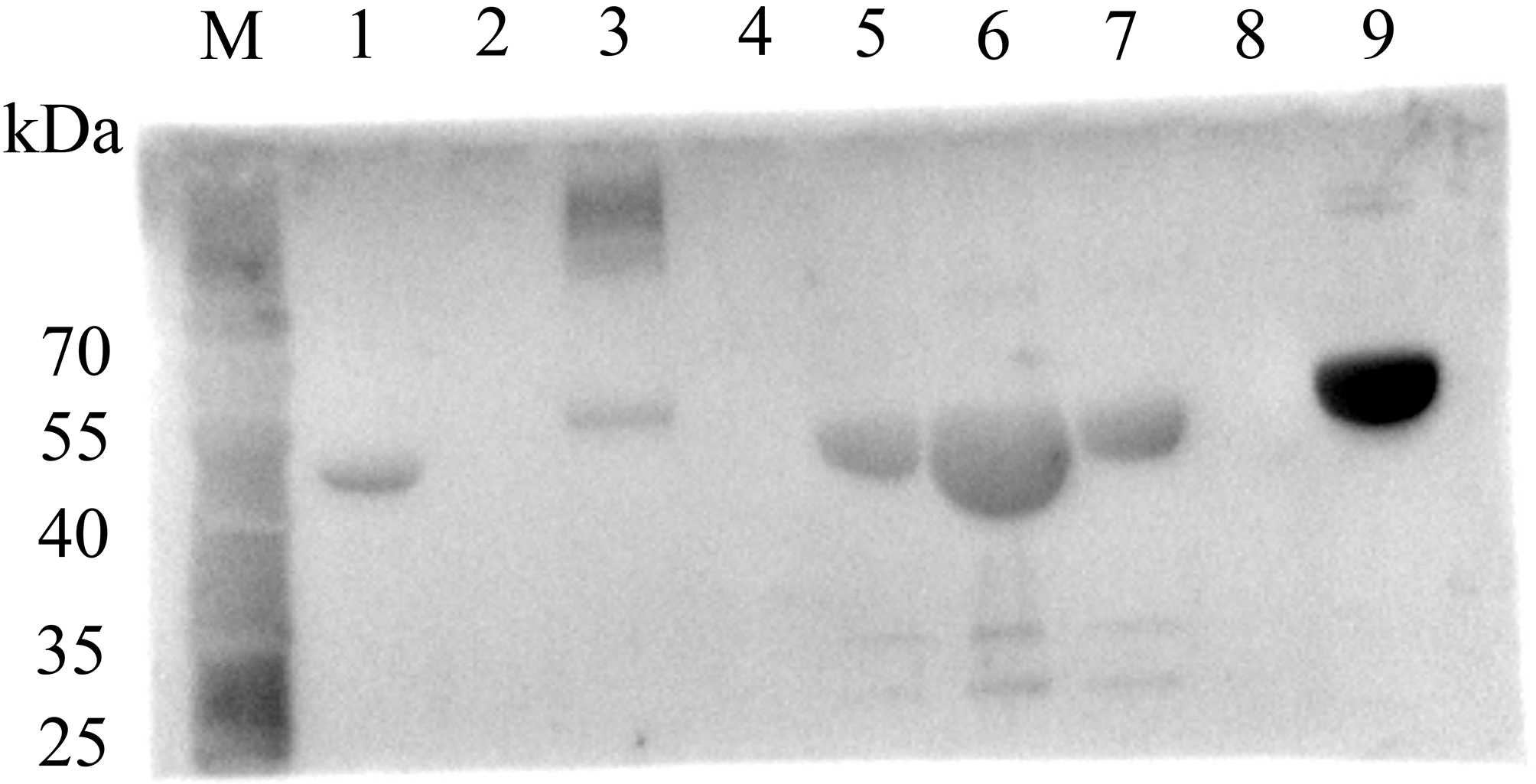
**

**Fig. S4.** *M. hyopneumoniae* proteins with fibronectin interaction analysis by far Western blot. Lane M, prestained protein mass marker. Lane 1, purified recombinant protein rNFOR. PVDF membrane with transferred Mhp NFOR protein incubated with fibronectin and the anti-fibronectin antibody. Lane 2, 4 and 8, BSA, PVDF membrane with transferred BSA (negative control) incubated with fibronectin and the anti-fibronectin antibody. Lane 3, purified recombinant protein rNOX. PVDF membrane with transferred Mhp NOX protein incubated with fibronectin and the anti-fibronectin antibody. Lane 5, 6, 7 and 9, purified recombinant protein rLAP. PVDF membrane with transferred Mhp LAP protein incubated with fibronectin and the anti-fibronectin antibody.
